# Suspended waveguide-enhanced near-infrared photothermal spectroscopy for ppb-level molecular gas sensing on a chalcogenide chip

**DOI:** 10.1038/s41377-026-02196-7

**Published:** 2026-02-17

**Authors:** Kaiyuan Zheng, Hanyu Liao, Fengbo Han, Xueying Wang, Yan Zhang, Jiaxin Gu, Pengcheng Zhao, Haihong Bao, Shaoliang Yu, Qingyang Du, Lei Liang, Chuantao Zheng, Wei Jin, Lijun Wang

**Affiliations:** 1 Department of Electrical and Electronic Engineering and Photonics Research Institute, The Hong Kong Polytechnic University, 999077 Hong Kong, China; 2 Photonics Research Center, The Hong Kong Polytechnic University Shenzhen Research Institute, 518071 Shenzhen, China; 3 Zhejiang Lab, 311121 Hangzhou, China; 4 State Key Laboratory of Integrated Optoelectronics, JLU Region, College of Electronic Science and Engineering, Jilin University, 130012 Changchun, China; 5 State Key Laboratory of Luminescence and Applications, Changchun Institute of Optics, Fine Mechanics and Physics, Chinese Academy of Sciences, 130033 Changchun, China

**Keywords:** Optical sensors, Optical spectroscopy

## Abstract

On-chip waveguide sensors have attracted significant attention recently due to their potential for high-level integration. However, so far, on-chip gas sensing based on traditional laser absorption spectroscopy has demonstrated low detection sensitivity, due to weak light-gas interaction over a limited interaction distance. On-chip photothermal spectroscopy (PTS) appears to be a powerful technique to achieve higher sensitivity; its performance is yet constrained to parts-per-million (ppm)-level due to a small fraction of evanescent field in the light-gas interaction zone and fast thermal dissipation through the solid substrate. Herein, we demonstrated suspended chalcogenide glass waveguide (ChGW)-enhanced PTS that overcomes these limitations, enabling highly sensitive parts-per-billion (ppb)-level molecular gas sensing. We fabricated a nanoscale suspended ChGW with a low loss of 2.6 dB/cm using a CMOS-compatible two-step patterning process. By establishing an equivalent PTS model to guide the optimization of the ChGW geometry, we achieved a 4-fold increase in the absorption-induced heat source power and a 10.6-fold decrease in the equivalent heat conductivity, resulting in a 45-fold enhancement in photothermal phase modulation efficiency over the non-suspended waveguides. Combining with a high-contrast waveguide facet-formed Fabry-Perot interferometer, we achieved an unprecedented acetylene detection limit of 330 ppb, a large dynamic range close to 6 orders of magnitude, and a fast response of less than 1 s. The overall system exhibits a noise-equivalent absorption coefficient of 3.8×10^−7^ cm^−1^, setting a new benchmark for photonic waveguide gas sensors to the best of our knowledge. This work provides a key advancement towards prototyping an integrated sensor-on-a-chip for highly sensitive and background-free photonic sensing applications.

## Abstract (web summary)

This work reports a novel nanophotonic platform based on a suspended chalcogenide glass waveguide for on-chip photothermal spectroscopy, enabling highly sensitive near-infrared molecular gas sensing at the parts-per-billion level.

## Graphical abstract

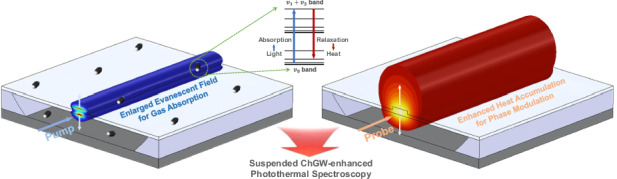

## Introduction

With the increasing demand of portable sensors for environmental monitoring and wearable healthcare^1,2^, on-chip photonic gas sensors have garnered significant attention due to their advantages in size, weight, power, and cost (SWaP-C)^3–5^. Most on-chip gas sensors reported so far are based on direct absorption spectroscopy (DAS), relying on the interaction of gas molecules with the evanescent field of a photonic waveguide^6–9^. They operate in the near-infrared (NIR) or mid-infrared (MIR) regions and have waveguide lengths on the order of centimeters, achieving detection sensitivity from hundreds to several ppm^10,11^. An alternative approach leverages a highly sensitive on-chip resonant cavity and detects the refractive index (RI) change in the presence of gas molecules. This method achieves sensitivity down to the ppm level but lacks specificity^12,13^. The limited sensitivity is due to the intrinsically short optical pathlength, residual fringing noise, and weak light-gas interaction, particularly in the NIR region.

Recent advances in photothermal spectroscopy (PTS) have proved itself to be a highly sensitive and selective technique for trace gas detection^14,15^. Gas molecules absorb a pump beam with a wavelength tuned to a specific gas absorption line, which generates heat through molecule collisions and changes the temperature distribution of the surrounding medium through heat conduction. PTS measures photothermally induced RI change by detecting the phase modulation of the probe beam, benefiting from both the high sensitivity of RI sensing and the high specificity of absorption spectroscopy^16–18^. PTS is recognized as a background-free technique, where phase measurement offers superior sensitivity, larger dynamic range, and reduced susceptibility to fringing noise compared to intensity-based DAS^15^. In recent years, PTS has transitioned from free-space systems with millimeter-scale mode field diameters (MFD) to fiber-optic platforms with micrometer-scale MFD and has achieved significantly enhanced sensitivity due to improved photothermal (PT) efficiency, which is inversely proportional to the MFD. Nanometer-scale waveguides could have a much smaller MFD, which would enable a higher PT efficiency and have progressed from theoretical designs to experimental demonstrations recently^19^. However, their performance is limited by the small fraction of evanescent field for light-gas interaction, resulting in a low level of heat source power due to absorption. In addition, rapid heat dissipation from the solid bottom-cladding material, such as silica (SiO_2_) with high thermal conductivity, reduces heat accumulation and impairs PTS sensing performance. Prior studies show that using lower-thermal-conductivity polymer bottom-cladding^20^ or introducing an air gap^21^ can mitigate heat leakage and improve light-gas overlap. However, there remains a lack of systematic guidance and quantitative evaluation on how to optimize these approaches for gas sensing, particularly regarding phase modulation amplification and thermal resistance. The relative performance of the suspended method compared to other thermal isolation strategies also remains unclear.

Herein, we present an on-chip suspended waveguide-enhanced PTS (SWE-PTS) to address the limitations of current photonic sensors. We theoretically establish an equivalent PT model to quantitatively evaluate the heat generation, conduction dynamics and PT modulation on a suspended ChGW surrounded by absorptive gas, which guides the optimization of the waveguide material, geometry and length to achieve optimal PT efficiency. Theoretical analysis reveals that the suspended ChGW demonstrates a nearly 2 orders of magnitude enhancement in PT efficiency compared to the traditional non-suspended one. We then fabricated a low-loss suspended ChGW with a 3-μm-thick air buffer zone (bottom cladding) by using a standard CMOS-compatible two-pattern membrane release process. Compared with solid SiO_2_ bottom cladding, the air buffer zone significantly enhanced the evanescent field, resulting in a larger optical absorption and thus higher heat source power. The thermal conductivity of air is more than 50 times smaller than that of SiO_2_, which promotes heat accumulation in the buffer zone and hence induces a large temperature variation. Such enhanced heat accumulation, coupled with the large thermos-optic coefficient of the ChGW material, enables significant enhancement of PT phase modulation and hence higher gas detection sensitivity. We further validate the capability of its gas molecular sensing capability of SWE-PTS by incorporating an on-chip Fabry-Perot interferometer utilizing the inherent reflections at the waveguide facets, enabling an integrated device for heat generation and detection simultaneously. As a result, our photonic waveguide gas sensor achieves unprecedented ppb-level gas detection for the first time in the NIR, which is particularly significant given the orders of magnitude weaker absorption in the NIR as compared to MIR, and a short chip length of only 1.2 cm. Photonics in the NIR is relatively mature and easy to integrate with light sources and detectors, paving the way for next-generation high-sensitivity, high-specificity, and large-scale integrated photonic sensors.

## Results

### Theoretical formulation

**Fig. 1:**
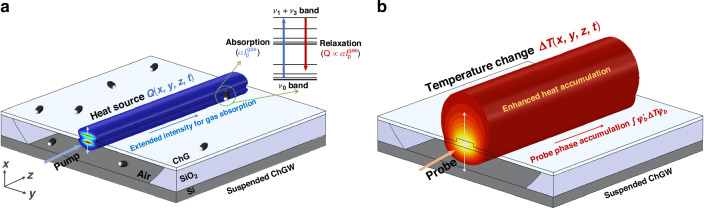
Principle of the SWE-PTS. **a** Heat generation by pump absorption. The pump beam exhibits a strong evanescent field in the gas region above and below the waveguide. The gas molecules absorb the pump energy and transit to higher energy level, which subsequently returns to the ground state via thermal relaxation and produces heat source ${Q}$. The profile of the heat source follows the evanescent pump intensity profile ${\psi }_{{\rm{p}}}^{* }{\psi }_{{\rm{p}}}$ as a two-petal shape in the air. The suspended waveguide design enhances the product of fractional power in the evanescent field and the mode group index, which is referred to as optical enhancement. The inset shows the exemplary energy levels of acetylene for the heat generation process. **b** Temperature-induced probe phase modulation. Due to the heat conduction, the temperature distribution ${\psi }_{{\rm{T}}}$ is no longer of a two-petal shape, where the *T* is approximately uniform in the ChG cross-section, and it extends and diminishes in the air due to their large differential thermal conductivity. The heat accumulation is improved by the suspended design, which prevents the rapid heat loss through the ChG and substrates. The probe beam experiences a phase change with respect to the spatial integration of optical and thermal mode field ${\psi }_{{\rm{b}}}^{* }{\psi }_{{\rm{T}}}{\psi }_{{\rm{b}}}$. The suspended design reduces the heat conduction and improves the thermo-optic effect as ${e}_{{\rm{eq}}}^{{\rm{TO}}}/{\kappa }_{{\rm{eq}}}$, which is referred to as thermal enhancement

The suspended waveguide uses air as the top and bottom cladding, a ridge structure as the core layer and a doped silicon (Si) as the substrate, as shown in Fig. 1a. A pump-probe configuration is used, where a wavelength-modulated pump beam, with its nominal wavelength tuned to a gas absorption line, propagates along the suspended ChGW with an evanescent field extending into the air cladding. Gas molecules absorb the pump energy in the evanescent field, resulting in heat generation due to the non-radiative relaxation. The corresponding heat source $Q(x,y,t)$ follows the intensity profile of the pump evanescent field in the air region. Since the pump wavelength is modulated around the gas absorption line, the heat generation is periodic and can be decomposed into a series of harmonics of the modulation frequency. The *n*^th^ harmonic heat source may be expressed as:

1 $${Q}_{{\rm{n}}}(x,y,t)={H}_{{\rm{n}}}\alpha C{I}_{{\rm{p}}}^{{\rm{gas}}}{\left(x,y\right)e}^{{\rm{jn}}{\rm{\omega }}{\rm{t}}}$$

where ${H}_{{\rm{n}}}$ is a harmonic coefficient depending on the depth of wavelength-modulation with respect to the gas absorption lineshape function^14^, $\alpha$ is the peak absorption coefficient of the analyte (for C_2_H_2_, *α* = 1.05 cm^-1^ at 1531.58 nm), $C$ is the gas concentration, $\omega$=2π*f* with *f* representing the pump modulation frequency. ${I}_{{\rm{p}}}^{{\rm{gas}}}(x,y)$ is the pump intensity distribution with superscript denotes the light intensity in the gas region. Taking the 2nd harmonic heat source as an example, *n* = 2, *H*_2_ = 0.343, which is obtained with an optimal modulation depth of 2.2 times the absorption linewidth^22^. The total heat source power ${P}_{{\rm{Q}}}$ due to absorption of the incident pump power ${P}_{{\rm{p}}}$ may be written as:

2 $${P}_{{\rm{Q}}}(t)=\iint {Q}_{2}{dxdy}={H}_{2}\alpha C{P}_{{\rm{p}}}\varGamma {e}^{{\rm{j}}2\omega {\rm{t}}}$$

3 $$\varGamma :=\frac{\iint {I}_{{\rm{p}}}^{{\rm{gas}}}{dxdy}}{{P}_{{\rm{p}}}}=\frac{{n}_{{\rm{gp}}}{\iint }_{{\rm{gas}}}\,\varepsilon {\left|{E}_{{\rm{p}}}\right|}^{2}{dxdy}}{{n}_{{\rm{gas}}}{\iint }_{{\rm{total}}}\,\varepsilon {\left|{E}_{{\rm{p}}}\right|}^{2}{dxdy}}=\frac{n_{\rm gp}}{n_{\rm gas}}\gamma_{\rm p}$$

here $\varGamma$ is the light-gas confinement factor that scales the interaction strength^23^, ${n}_{{\rm{gp}}}$ is the group index of the pump mode in the suspended ChGW, ${n}_{{\rm{gas}}}$ is the RI of gas, $\varepsilon (x,y)$ and ${E}_{{\rm{p}}}(x,y)$ are respectively the permittivity and the electric field distribution of the pump. According to Eqs. (2–3), the incident pump power ${P}_{{\rm{p}}}$ is converted to the heat source power $|{P}_{{\rm{Q}}}|$ scaled by the unit-less factor $\varGamma$, which benefits from the pump group index ${n}_{{\rm{gp}}}$ and the fraction of the pump evanescent field ${\gamma }_{{\rm{p}}}$. The suspended design of ChGW significantly improves the light-gas interaction through a higher evanescent field in gas, hence a higher heat power ${P}_{{\rm{Q}}}$^24,25^.

The heat source $Q$ leads to a change of temperature distribution$\,\Delta T(x,y,t)$ across the waveguide cross-section through heat conduction, which can be detected by a probe beam in the form of phase modulation. Figure 1b shows the resulting temperature change for the pump modulation frequency of *f* = 1 kHz and *n* = 2. The suspended design creates a thermal buffer zone before the heat reaches the Si substrate, which enables heat accumulation around the probe mode and hence a larger PT phase modulation. To quantify the improvement of heat accumulation by suspended design, we introduce an equivalent heat transfer model by decomposing the temperature change $\Delta T$ into a product:

4 $$\Delta T\left(x,y,t\right)={T}_{{\rm{eq}}}{{\psi }_{{\rm{T}}}\left(x,y\right)e}^{{\rm{j}}2{\rm{\omega }}{\rm{t}}}$$

where ${\psi }_{{\rm{T}}}$ is the temperature spatial mode profile capturing the geometric dependence of the temperature change, ${T}_{{\rm{eq}}}$ is the equivalent temperature amplitude scaling the mode profile. This separable form arises since the fixed de-modulation frequency and fixed pump wavelength define a specific mode ${\psi }_{{\rm{T}}}(x,y)$ for the heat transfer in the waveguide cross-section. For SWE-PTS, we are only interested in the overlap between the probe field ${\psi }_{{\rm{b}}}$ and the temperature field ${\psi }_{{\rm{T}}}$. Thus, we normalize ${\psi }_{{\rm{T}}}$ by projecting it onto the probe mode under a weighted inner product:

5 $${\iint }_{{\rm{total}}}\,{\psi }_{{\rm{b}}}^{* }{\psi }_{{\rm{T}}}{\psi }_{{\rm{b}}}{dxdy}{\rm{:= }}1$$

here ${\psi }_{{\rm{b}}}$ is the probe mode field. Equation (5) ensures unit coupling strength between ${\psi }_{{\rm{T}}}$ and the PT phase modulation on mode ${\psi }_{{\rm{b}}}$. Based on Eqs. (4–5), the heat transfer can be analytically solved in integration form for constant temperature boundaries (Supplementary Note 1). This approach permits analysis of the system’s macroscopic behavior without resolving spatial transients, i.e., the equivalent temperature amplitude ${T}_{{\rm{eq}}}$:

6 $${T}_{{\rm{eq}}}={\iint }_{{\rm{total}}}\,{\psi }_{{\rm{b}}}^{* }\widetilde{T}{\psi }_{{\rm{b}}}{dxdy}=\frac{{P}_{Q}}{j2{\omega }_{m}{C}_{{eq}}-{\kappa }_{{eq}}}$$

where we have introduced equivalent volumetric heat capacity ${C}_{{eq}}$ and heat conductivity ${\kappa }_{{\rm{eq}}}$ to conclude the whole heat transfer characteristics:

7 $${\kappa }_{{\rm{eq}}}={\iint }_{{\rm{total}}}\,\nabla \cdot \left(\kappa (x,y)\nabla {\psi }_{{\rm{T}}}\right){dxdy}$$

8 $${C}_{{eq}}=\,{\iint }_{{total}}\,C(x,y){\psi }_{T}{dxdy}$$

According to Eqs. (7–8), ${C}_{{eq}}$ and ${\kappa }_{{\rm{eq}}}$ capture the overall thermal convection and conduction behavior in the x-y plane experienced by ${\psi }_{T}$. The equivalent temperature ${T}_{{\rm{eq}}}$ is actually the temperature value probed by the probe mode ${\psi }_{{\rm{b}}}^{* }{\psi }_{{\rm{b}}}$. To achieve maximum phase modulation efficiency, we choose modulation frequency ${\omega }_{m}=1{kHz}\ll {C}_{{eq}}/2{\kappa }_{{eq}}$ to obtain maximum ${T}_{{\rm{eq}}}=-{P}_{Q}/{\kappa }_{{eq}}$, and the error of this approximation is only 0.5% in our case. Hence, the ${T}_{{eq}}$ depends on the core-layer material thermal conductivity and the suspended ChGW geometric parameters. For molecular gas sensing, we measure the phase modulation $\Delta {\varphi }_{{\rm{b}}}$ on the probe beam induced by the temperature change through thermal-optic effect (TOE), which could be expressed as:

9 $$\Delta {\varphi }_{{\rm{b}}}\approx -\frac{2\pi {n}_{{\rm{gb}}}L}{{\lambda }_{{\rm{b}}}}\frac{{P}_{{\rm{Q}}}}{{\kappa }_{{\rm{eq}}}}\left({e}_{{\rm{ChG}}}^{{\rm{TO}}}{\iint }_{{\rm{ChG}}}\,{\psi }_{{\rm{b}}}^{* }{\psi }_{{\rm{T}}}{\psi }_{{\rm{b}}}{dxdy}+{e}_{{\rm{gas}}}^{{\rm{TO}}}{\iint }_{{\rm{gas}}}\,{\psi }_{{\rm{b}}}^{* }{\psi }_{{\rm{T}}}{\psi }_{{\rm{b}}}{dxdy}\right){e}^{{\rm{j}}2{\rm{\omega }}{\rm{t}}}$$

here ${\lambda }_{{\rm{b}}}$ is the probe wavelength ${{n}}_{{\rm{gb}}}$ the group index of the probe mode, ${L}$ the waveguide length, and ${e}^{{\rm{TO}}}={dn}/{dT}$ is the TO coefficient with subscript ChG, and gas denotes the core-layer ChG and outside gas. According to Eq. (9), the PT phase modulation is proportional to ${\kappa }_{{\rm{eq}}}^{-1}$. As compared to the conventional waveguide employing SiO_2_ as the bottom cladding, the suspension design reduces the equivalent thermal conduction ${\kappa }_{{\rm{eq}}}$ and hence a much higher heat accumulation quantified by ${T}_{{\rm{eq}}}$.

To compare the SWE-PTS among different suspended geometric parameters, including the rib width (*w*), height (*h*) and rib ratio (*k*), we define a normalized PT phase modulation efficiency:

10 $${k}^{* }\left(w,h,k\right){\rm{:= }}\frac{\Delta {\varphi }_{{\rm{b}}}}{\alpha C{P}_{{\rm{p}}}L}\propto \varGamma \frac{{e}_{{\rm{eq}}}^{{\rm{TO}}}}{{\kappa }_{{\rm{eq}}}}$$

here ${k}^{* }\left(w,h,k\right)$ is defined as the normalized PT phase modulation efficiency, ${e}_{{\rm{eq}}}^{{\rm{TO}}}$ represents the terms in the parenthesis in Eq. (9) and is regarded as the equivalent thermo-optic coefficient. According to Eq. (10), the suspended waveguide achieves superior PT phase modulation efficiency through dual optimization mechanisms: optical enhancement via enlarged optical absorption ($\varGamma$), and thermal enhancement via combining larger equivalent thermal resistance ($1/{\kappa }_{{\rm{eq}}}$) and amplified thermo-optic response (${e}_{{\rm{eq}}}^{{\rm{TO}}}$). Numerical calculation reveals that suspending a ChG core-layer with a bottom-cladding thickness of several microns can improve the ${k}^{* }$ value by nearly 2 orders of magnitude. This remarkable improvement primarily stems from a 3−5 times increment in $\varGamma$ and 10−30 times larger ${e}_{{\rm{eq}}}^{{\rm{TO}}}/{\kappa }_{{\rm{eq}}}$, making it possible for highly sensitive gas detection on a short-length chip. More details of SWE-PTS refer to Supplementary Note 1.

### Optimization of suspended ChGW

The waveguide material and geometry work together to determine the optical and thermal properties and hence the SWE-PTS performance. The selection criteria of the material, especially the core-layer, include large TOE, large thermal expansion effect (TEE), and low *κ* coefficient. To facilitate comparison, we use a PT figure-of-merit (FOM) of *n*×(TOE + TEE)/*κ*^26,27^. ChG exhibits an FOM of 2.87×10^–4^ m/W, which is 2–3 orders of magnitude larger than Si, silicon nitride (Si_3_N_4_), and lithium niobate (LiNbO_3_)^20^. Therefore, ChG is selected as the core-layer material in our suspended waveguide design. Specifically, we choose the ChG composition Ge_28_Sb_12_Se_60_, also commercially known as IG5, as it is resistant to both photo-darkening effect and oxidation in the ambient environment compared with other arsenic-containing compositions.

**Fig. 2:**
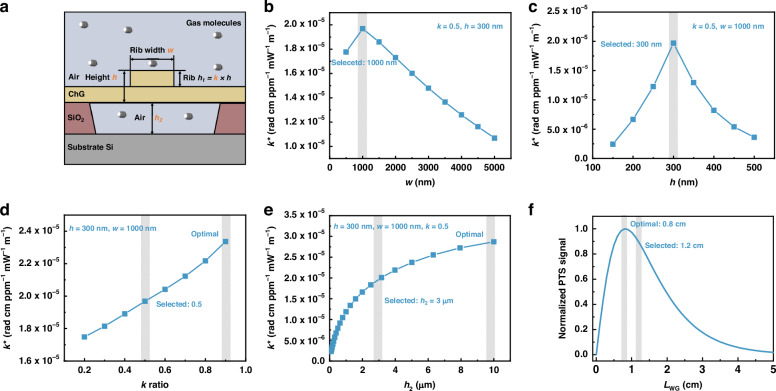
Optimization of the suspended ChGW for enhanced SWE-PTS. **a** Schematic of the suspended waveguide with air serving as the top and bottom claddings. The phase modulation efficiency as a function of **b** rib width (*w*), **c** core-layer height (*h*), **d** rib ratio (*k*), and **e** bottom buffer zone thickness (*h*_2_). **f** The overall PTS signal for varying waveguide length

To optimize the suspended ChGW structure, we compare the phase modulation efficiency ${k}^{* }$ for different waveguide parameters using COMSOL Multiphysics. As shown in Fig. 2a–e, the optimal parameters are *w* = 1000 nm, *h* = 300 nm, *k* = 0.9, and *h*_2_ = 10 μm. Considering that a thin slab with a large rib ratio *k* = *h*_1_/*h* is mechanically fragile, which may lead to collapse of the entire suspended structure, we decide to select the set of *w* = 1000 nm, *h* = 300 nm, *k* = 0.5, and *h*_2_ = 3 μm for a robust waveguide design without compromising too much of its performance. The waveguide length ${L}_{{\rm{wg}}}$ is determined by balancing the PT phase modulation with transmission loss. There is a trade-off, as the PT phase modulation $\Delta {\varphi }_{{\rm{b}}}$ generally increases with the waveguide length. However, a long waveguide diminishes the fringe contrast ($v$) of the waveguide interferometer due to increased transmission loss, and thus reduces the efficiency of phase-to-intensity conversion and the overall PTS signal. Figure S3 shows the dependence of normalized $\Delta {\varphi }_{{\rm{b}}}$ and $v$ on the waveguide length. The variation of the PTS signal with the ChGW length is shown in Fig. 2f, which indicates an optimal length of ~0.8 cm. In this setup, the actual ChGW length is 1.2 cm, which remains ~90% of the optimal value (Details in Supplementary Note 3).

### Comparison with non-suspended ChGW

**Fig. 3:**
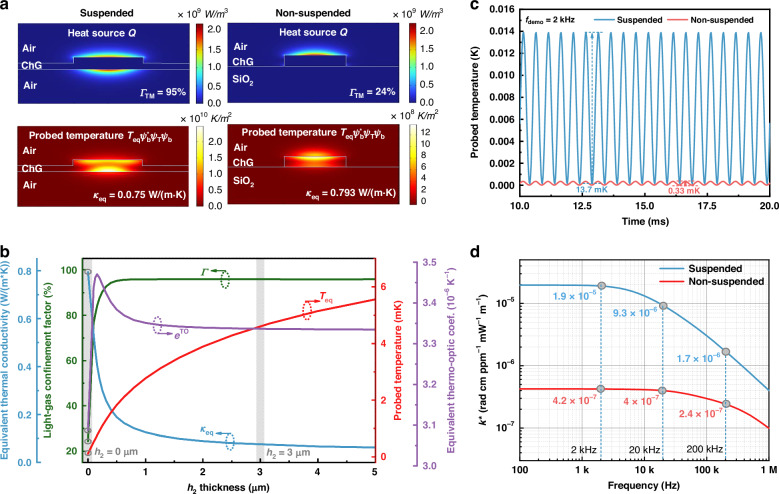
Enhancement factor evaluation. **a** Comparison of optical (upper panel) and thermal (lower panel) confinements of the suspended and non-suspended waveguide structures. The *Γ* value of the suspended waveguide is ~4 times larger than that of the non-suspended one, and$\,{\kappa }_{{\rm{eq}}}$ is ~10.6 times smaller than the non-suspended one. **b** Dependence of the $\varGamma$, ${e}_{{\rm{eq}}}^{{\rm{TO}}},\,{\kappa }_{{\rm{eq}}}$ and ${T}_{{\rm{eq}}}$ on the t*h*ickness of the air buffer zone *h*_2_. The circled points at *h*_2_ = 0 μm correspond to the non-suspended structure. **c** Time-domain simulation at demodulation frequency of 2 kHz. The temperature variation is ~42 times larger than the non-suspended one. **d** Frequency^-^domain simulation. At 2 kHz, k^*^ value is nearly 45 times larger than the traditional non-suspended one

We then evaluated the optical and thermal enhancement in the optimized suspended ChGW over the non-suspended case through numerical simulation. Here, the bottom-cladding is air for the suspended waveguide and SiO_2_ for the non-suspended one^28,29^. Numerical calculation is performed with dimensions of *w* = 1000 nm, *h* = 300 nm, and *k* = 0.5, with a 3-μm-thick air bottom-cladding as the buffer zone for the suspended one, and *w* = 1000 nm, *h* = 300 nm for the non-suspended strip one. Both TE and TM modes are supported in the suspended ChGW, but only the TM mode is selectively excited in this case due to its larger evanescent field, which would lead to stronger light-gas absorption. Figure 3a shows the heat source due to gas absorption of the pump field (${\psi }_{{\rm{p}}}^{* }{\psi }_{{\rm{p}}}$) and the probed thermal field (${T}_{{\rm{eq}}}{\psi }_{{\rm{b}}}^{* }{\psi }_{{\rm{T}}}{\psi }_{{\rm{b}}}$), respectively, with their integrals corresponding to the optical enhancement ($\varGamma$) and thermal enhancement (${e}_{{\rm{eq}}}^{{\rm{TO}}}/{\kappa }_{{\rm{eq}}}$) according to Eqs. (2) and (6), respectively. With a pump wavelength of ~1531 nm, the pump field in TM_0_ mode exhibits a dual-sided evanescent field in air, resulting in a much higher $\varGamma$ of 95% than the non-suspended one of 24%^30^. For the thermal enhancement, the suspension design creates a thermal buffer zone, reducing the equivalent thermal conductivity ${\kappa }_{{\rm{eq}}}$ from 0.793 to 0.075 W/(m ∙ K) (Methods). Figure 3b shows the dependence of the $\varGamma$, ${e}_{{\rm{eq}}}^{{\rm{TO}}},\,{\kappa }_{{\rm{eq}}}$ and ${T}_{{\rm{eq}}}$ with the increasing thickness of the air buffer zone *h*_2_ from 0 to 5 μm. Compared to the non-suspended ChGW structure (*h*_2_ = 0 μm), the values of $\varGamma$, ${e}_{{\rm{eq}}}^{{\rm{TO}}}$, ${\kappa }_{{\rm{eq}}}$ and ${T}_{{\rm{eq}}}$ for the case of 3-μm-thick buffer zone are enhanced by a factor of 4, 1.08, 10.6 and 42, respectively.

To illustrate the improvement in the PT response, we calculate the time-domain temperature modulation at the demodulation frequency of 2 kHz (*n* = 2) and the results are shown in Fig. 3c. The probed temperature change in the suspended structure increases from 0.33 mK to 13.7 mK, approximately 42 times that of the non-suspended one. This clearly shows that good thermal isolation promotes the rapid rise and fall of the thermal dynamic homeostasis of the PT effect^14,31^. The PT phase modulations of the suspended and non-suspended ChGW are determined by steady-state frequency-domain simulations and are shown in Fig. 3d. At a demodulation frequency of 2 kHz, a significant k^*^ enhancement of 45 times is achieved, which evidences that the suspended structure is a better option for PTS than the non-suspended one. Detailed comparisons are provided in Supplementary Note 4.

### The suspended ChGW

The suspended ChGW is fabricated following a CMOS-compatible process on a 6-inch silicon wafer with a 3-μm-thick oxide cladding^32^. A novel two-step patterning is utilized in this work to fabricate the suspended ChGW with low transmission loss and high mechanical stability. In the first patterning, holes are dry-etched on the evaporated thin film and are also used for subsequent wet etching. Ridge waveguide structures with optimized parameters are then formed using the second patterning. The process is completed by removing the underlying silicon oxide layer with hydrofluoric acid to form a suspended ChGW. Detailed processes refer to Methods and Supplementary Note 5.

**Fig. 4:**
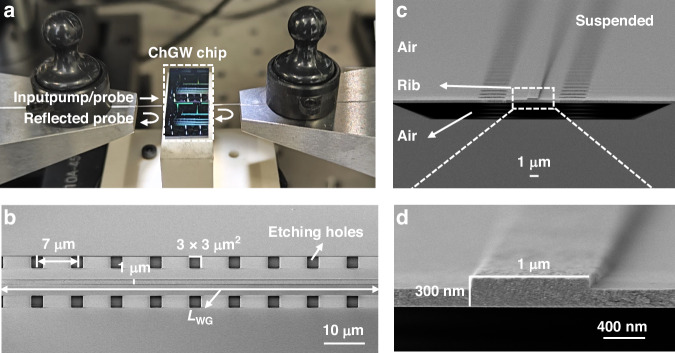
Suspended ChGW fabrication. **a** End-face coupling platform between the tapered fiber and the suspended ChGW chip with a coupling loss of ~8 dB/facet. Such a loss is mainly due to the mismatch of the optical mode overlaps and can be optimized by exploiting inverse-tapered coupling. SEM images of the suspended chip from **b** top view with 3 × 3 μm^2^ channels for membrane release. **c** Cross-section view and **d** Zoom-in view

The scanning electron microscope (SEM) images of the fabricated suspended ChGW are shown in Fig. 4b–d. The top view of the square openings in Fig. 4b depicts 3 × 3 μm^2^ channels used for membrane release, which also enables sufficient gas flow for light-gas interaction along the waveguide. The transmission loss of the ChGW is evaluated to be ~2.6 dB/cm (Supplementary Note 3)^33^. Figure 4c, d show the SEM cross-section views of the suspended ChGW with a rib height of ~300 nm and a width of ~1000 nm. After fabricating the suspended ChGW, a tapered silica fiber (for delivering the pump and probe beams into the ChGW) is end-faced coupled into one end of the ChGW. As shown in Fig. 4a, the inherent reflections at the waveguide/air facets form an F-P cavity, which is used as an optical interferometer to convert the PT phase modulation into detectable intensity variations (Supplementary Note 2)^14,34^.

### Characterization of on-chip F-P cavity

**Fig. 5:**
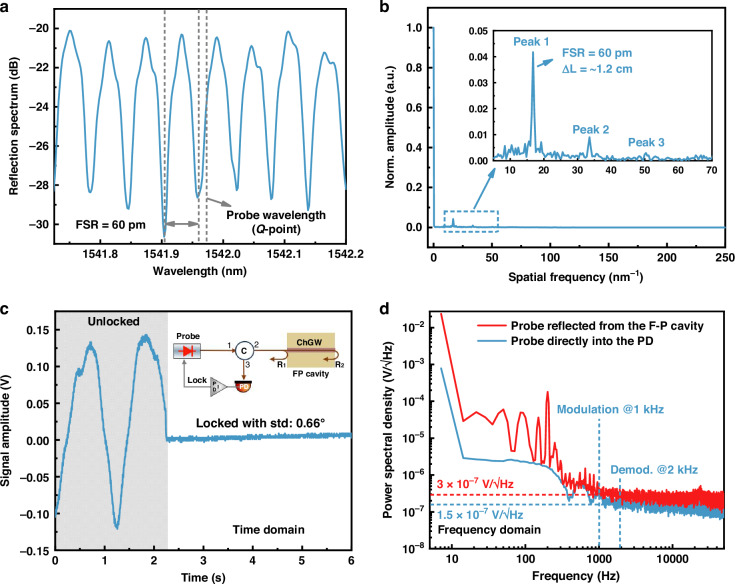
F-P cavity characterization. **a** Reflection spectrum of the F-P cavity and **b** its FFT result. Inset shows three harmonic peaks caused by multiple reflections within the interface of waveguide/air, confirming the existence of the F-P cavity. **c** Servo-locking process of the F-P cavity at the quadrature point to stabilize the interferometer. Inset shows the schematic of the F-P cavity of the probe beam. Detailed set-up is shown in Fig. 6a. **d** F-P cavity noise assessment using the PSD measurement with an ESA, showing low-noise performance over 1 kHz

As depicted in Fig. 5a, the reflection spectrum of the F-P cavity is measured using a broadband amplified spontaneous emission (ASE) source and an optical spectrum analyzer (OSA) with a wavelength resolution of 10 pm. The free spectral range (FSR) and fringe contrast of the interference spectrum around 1542 nm are approximately ~60 pm and ~8 dB, respectively. The FSR is determined using *λ*^2^/2*n*_*m*_*L*, where *n*_m_ is the effective mode RI of the probe beam (at ~1542 nm), and *L* is the cavity length (~1.2 cm). The resultant *n*_m_ is approximately 1.6, which is closer to the simulated TM_0_ mode (*n* = 1.4) than the TE_0_ mode (*n* = 2.2), verifying that a TM_0_-dominated mode is transmitted along the suspended ChGW. Fast Fourier transform (FFT) of the measured reflection spectrum reveals several minor harmonic peaks, as shown in Fig. 5b, indicating some residual higher-order reflections may exist in the F-P cavity. More details refer to Supplementary Note 7.

Figure 5c presents the servo-locking process of the F-P cavity around the quadrature point to stabilize the interferometer using a fiber laser as probe source. The servo amplitude stabilizes immediately after the servo stabilization is applied, and the standard deviation of the phase fluctuation is measured to be ~0.66°. We then examine the F-P cavity noise with results depicted in Fig. 5d. The noise level is characterized by measuring probe power spectral density (PSD) on the photodetector (PD) output using an electrical spectrum analyzer (ESA). For the first case with a probe beam directly into the PD without the F-P cavity (blue line), PSD reveals the relative intensity noise (RIN) of the probe laser. We then measured the PSD after the probe beam reflected from the F-P cavity (red line), where the power level of the PD input is the same as in the first case. The fluctuations caused by environmental perturbations (e.g., temperature drift, mechanical instability) are typically low-frequency (below 300 Hz). We employed a servo-loop system to compensate for these low-frequency noises, ensuring the F-P interferometer remains stable at the quadrature working point. For modulation frequencies above 1 kHz, the noise level is small, and thus a pump modulation frequency of 1 kHz (with demodulation at 2 kHz) is used in the following experiments. Notably, the PSD exhibits a twofold increase at modulation frequencies above 1 kHz compared to the direct PD measurement. This difference is mainly attributed to the phase noise from the fiber laser with a 2.4-cm-long optical path difference^35^.

### On-chip gas sensing

**Fig. 6:**
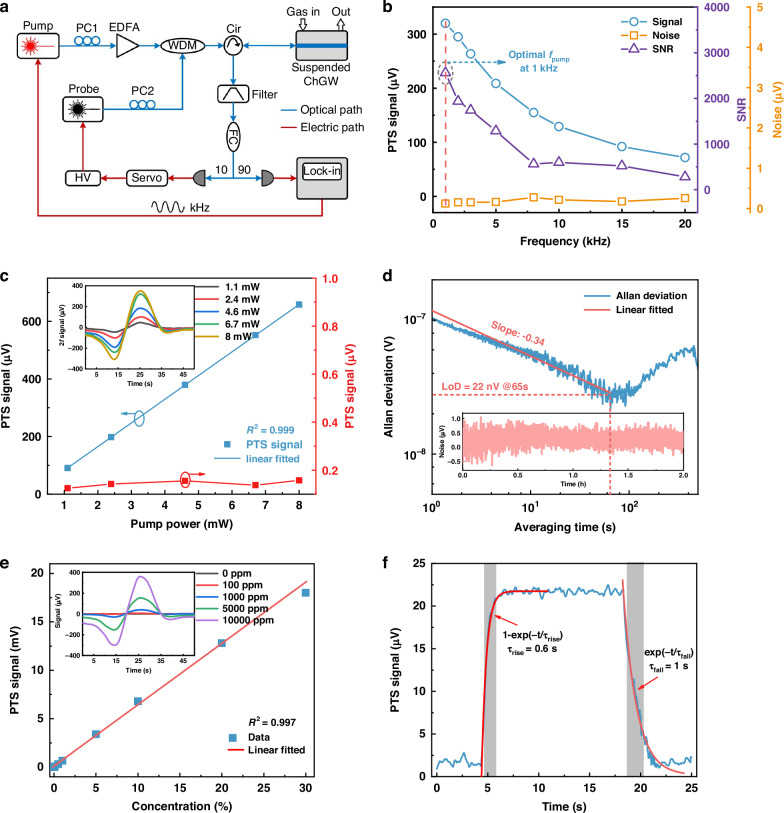
Gas sensing performance. **a** Experimental set-up of the SWE-PTS. EDFA: erbium-doped fiber amplifier, Cir: optical circulator, FC: fiber coupler, PD: photodetector. **b** Measured PTS signal amplitude, standard deviation (std) of the noise and SNR as a function of pump modulation frequencies. **c** Detected 2 *f* signal amplitude at *f* = 1 kHz and std. of the noise as functions of the pump power. Inset are the 2 *f* signals when the pump wavelength is tuned across the C_2_H_2_ lines. **d** Allan-Werle plot based on the noise data over a period of 2 h, which is shown in the inset. For the noise measurement, the lock-in time constant is 100 ms, corresponding to 0.94 Hz detection bandwidth. **e** Dynamic range evaluation with peak-to-peak value of the 2 *f* signal as a function of gas concentrations. The inset shows the 2 *f* signals for N_2_, 100 ppm, 1000 ppm, 0.5%, and 1% C_2_H_2_ when the pump power is set at 8 mW. The detection bandwidth is 0.094 Hz. **f** Response time evaluation. The PTS signal amplitude rises exponentially to a flat level following 1−exp(−*t*/*τ*_rise_) with the rise time *τ*_rise_, and then follows an exponential decay in the form of exp(−*t*/*τ*_fall_) with the rise time *τ*_fall_. At ∼4 s, 330 ppm C_2_H_2_ gas is loaded into the gas cell at a flow rate of 200 sccm, which is prefilled with N_2_. At ∼19 s, N_2_ is refilled into the gas cell at the same flow rate

The gas sensing performance of the SWE-PTS sensor is evaluated with the experimental setup shown in Fig. 6a. Both pump and probe beams are adjusted to TM_0_ mode using two polarization controllers (PCs). The wavelength of the distributed feedback (DFB) pump laser is sinusoidally modulated, with the center wavelength tuned to the P(11) absorption line of C_2_H_2_ at 1531.58 nm. As indicated in Fig. 5a, a probe beam from a narrow-linewidth (~1 kHz) fiber laser with its center wavelength tuned to the quadrature point (*Q*-point) at ~1541.955 nm is used to maximize the phase-to-intensity conversion efficiency. The reflected probe beam from the ChGW is split into two parts: one is into a servo-loop with proportion-integration-differentiation (PID), and low-pass filter components for servo-locking, and the other is for phase demodulation using a lock-in amplifier (LIA) to extract the 2 *f* signal. For trace gas detection, C_2_H_2_ samples with different concentration levels are prepared by mixing C_2_H_2_ with N_2_ at different flow-rate ratios.

Figure 6b shows the dependence of the PTS signal, noise level and measured signal-to-noise ratio (SNR) on the pump modulation frequency. The PTS signal decreases with increasing modulation frequency, and the 3-dB roll-off frequency is about ~8 kHz. The noise is measured by filling the gas cell with N_2_, and the maximum SNR is achieved around 1 kHz, hence we select 1 kHz in subsequent experiments. We validate the sensor performance by increasing the pump power inside the ChGW, as shown in Fig. 6c. A linear relationship between the PTS signal and input pump power is obtained with an *R*^2^ of 0.999. To further assess the detection limit of the SWE-PTS sensor, Allan-Werle analysis is conducted based on the noise data with N_2_ filling into the gas cell. As shown in Fig. 6d, the minimum Allan deviation is achieved at an averaging time of 65 s, where the lowest noise level is estimated to be ~22 nV, corresponding to a detection limit of 330 ppb for C_2_H_2_ detection. This yields a noise-equivalent absorption (NEA) of 3.8 × 10^−7^ cm^−1^, and a normalized noise-equivalent absorption (NNEA) of 9.9 × 10^-9^ cm^-1^ ∙ W∙Hz^-1/2^.

Figure 6e shows the peak-to-peak values of the 2 *f* signal as functions of C_2_H_2_ concentrations from 0 to 30%. The 2 *f* signal increases approximately linearly with the gas concentration, giving a dynamic range of nearly 6 orders of magnitude (∼9.1×10^5^). The response time is tested by sequentially filling the gas cell with N_2_, 330 ppm C_2_H_2_, and then N_2_ at a flow rate of 200 standard cubic centimeters per minute (sccm), while the pump wavelength is fixed at 1531.58 nm. Figure 6f shows the real-time recording of the PTS signal. The response time, specifically the time required for the response reaching 90% (i.e., rise time) and descending to 10% (*i.e*., fall time) of the maximum PTS signal, is less than 1 s. Such a response time is primarily limited by the gas exchange time, which is largely dependent on the volume of the gas chamber (~0.5 mL). Faster response can be obtained by increasing the inlet pressure difference or using a pump at the outlet; also, an on-chip microfluidic channel cell would certainly help reduce it to ~ms scale^36,37^.

## Discussion

In summary, we have reported a novel suspended ChGW nanophotonic platform for highly sensitive on-chip PTS gas molecular sensing. Theoretical and experimental results verified its superiority by improving the efficiency of heat generation and heat accumulation. Guided by an equivalent PT phase modulation model, we developed an optimized suspended ChGW that exhibits a nearly four times higher heat source power and a 10.6 times lower equivalent heat conductivity as compared to conventional strip waveguides. These factors work together to achieve 42 times higher probed temperature variation, leading to 45-fold larger PT phase modulation. We successfully demonstrated high-sensitivity C_2_H_2_ sensing with a detection limit of 330 ppb, a dynamic range spanning 6 orders of magnitude, and a fast response of less than 1 s. These comprehensive performance advantages pave the way for high-sensitivity, high-selectivity, wide dynamic range, rapid response, and ultracompact on-chip gas sensors, representing a significant step forward in the development of fully integrated photonic sensors.

**Fig. 7:**
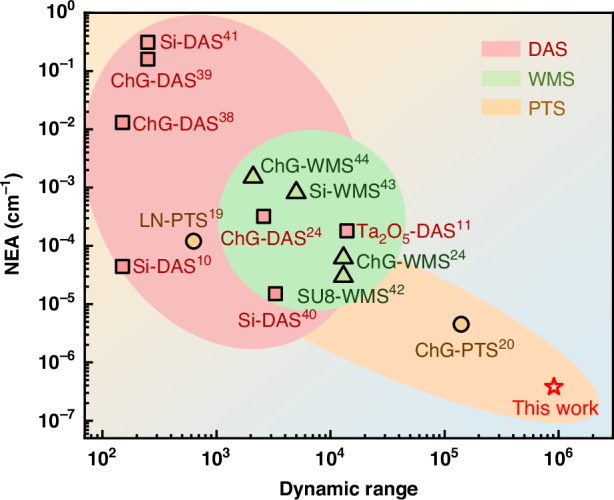
Performance comparison. Comparison of the SWE-PTS sensor with the state-of-the-art waveguide sensors in terms of NEA and dynamic range. DAS: direct absorption spectroscopy. WMS wavelength modulation spectroscopy, a variation of DAS. Si silicon, SU8 polymer photoresist, Ta_2_O_5_ tantalum pentoxide, LN lithium niobate

To benchmark the performance metrics of the reported SWE-PTS, we compare them with the representative state-of-the-art waveguide sensors, as shown in Fig. 7^10,11,13,19–21,24,38–44^. Detailed comparisons are provided in Supplementary Note 9. These sensors cover different analyte gases, system configurations and parameters. Here, we focus on comparing the sensor performance in terms of NEA and dynamic range. With a 1.2-cm-long suspended waveguide, the SWE-PTS sensor achieves NEA of 3.8 × 10^-7^ cm^-1^, representing an improvement of 1−4 orders of magnitude over existing waveguide sensors. The dynamic range reaches nearly 6 orders of magnitude, surpassing prior on-chip gas sensing technologies by more than 2 orders of magnitude. These remarkable results indicate that our sensor is the first photonic waveguide sensor capable of achieving ppb-level sensitivity in the NIR range. This is particularly significant given the orders of magnitude weaker absorption characteristics of the NIR compared to the MIR, as well as the relatively short interaction length of only 1.2 cm on a nanophotonic chip.

Better performance could be realized by further optimizing the sensor parameters. The transmission loss of ~2.6 dB/cm in this suspended ChGW primarily results from scattering due to the waveguide sidewall roughness. To minimize surface roughness, a waveguide thermal reflow process prior to suspension has been proven as an effective approach^45^. It is with great potential that the waveguide loss can be reduced to ~1 dB/cm, which potentially translates into an improved detection limit of less than 100 ppb. Additionally, ChG possesses an ultrawide transmission range from 1 to 16 μm, which would allow the detection of multiple molecular species in the NIR and MIR, including greenhouse gases and volatile organic compounds (Supplementary Note 8)^46^. Such an SWE-PTS system is readily extended into the MIR range with 2−3 orders of magnitude performance improvement using the strongest molecular absorption. Moreover, the extension of on-chip SWE-PTS to solid and liquid analysis is straightforward. Planar suspended platforms enable the investigation of photothermal, optomechanical, and optoacoustic effects in solid and liquid media, thereby significantly advancing the fields of interface optics^1^ and chemical reaction dynamics^2^. We also foresee the developed on-chip SWE-PTS as a highly potent technology for environmental monitoring^47^, health screening^48^, and wearable device applications^49^.

## Methods

### Suspended ChGW fabrication

A 6-inch CMOS-compatible fabrication line was used for suspended ChGW with a two-step patterning process. In the first step, the sacrificial silicon dioxide and ChG film were deposited onto the thermally oxidized silicon wafer by chemical vapor deposition and thermal evaporation, respectively. In addition, arrays of micro-holes were patterned and dry-etched by an inductive-coupled plasma (ICP) etcher for the final membrane release. The resist is then removed by soaking into (1-methyl-pyrodine) NMP and isopropanol (IPA) with the aid of ultrasonication. The second step involves the precise registration of the ChG rib waveguide between the micro-hole array. The rib waveguides were subsequently etched in ICP, and the corresponding photoresist was stripped. The final step involves immersing the waveguide in 5% HF, undercutting the thermally oxidized silicon through the micro-holes prepared in the first layer. This etching process is meticulously timed and controlled to prevent over-etching and structural damage. More details refer to Supplementary Notes 5 and 6.

### Fabrication tolerance and scalability estimation

To evaluate the fabrication tolerance, the cross-section metrology shows deviations of approximately 20–30 nm in waveguide width *w*, and around 3 nm in core-layer height *h*. The resulting fractional change in and hence the PTS SNR can be estimated with the aid of Fig. 2, where the overall performance variation is typically <1%. Among these geometric parameters, the core-layer height is the most sensitive and therefore requires more precise control during fabrication. The scaling to a multi-species gas detection sensor chip can be made with waveguide arrays, aiming at various detection wavelengths. Taking advantage of the CMOS technology, reliable, wafer-scale production is possible. The biggest challenge is the undercladding BOX removal process. It requires dipping the wafer into HF solution for precise time control. In addition, rinsing and cleaning may include the use of a critical point drier that helps retain large area suspended membrane without suffering through the high surface tension of water during drying.

### Gas cell and gas delivery system

The ChGW is sealed by a 3D-printed gas cell with two ports for gas exchange. One face is milled to form a downward-facing chamber and then inverted to cap the waveguide chip, defining a dimension of 1×1×0.5 cm^3^. Sample gas is delivered via 2 mm inner-diameter clear silicone tubing to the gas cell. Upstream of the cell, a 1.2 m straight run is included to ensure thermal equilibration and eliminate Joule-Thomson cooling effects. Using a simple convective heat-transfer estimate, for a pressure drop ~2 bar and a flow rate of 200 sccm, the characteristic thermal equilibration length is only ~3.5 cm and the Peclet number *Pe* ~ 10^-2^ ≪ 1, so the heat convection can be ignored, and the gas region can be considered at ambient temperature upon entering the cell. Inside the gas cell, the ChG waveguide is placed at the bottom of the gas chamber, approximately 3.5 mm below the jet axis of the gas inlet. The inlet port is positioned at the geometric center of the ceiling to avoid a focused jet directly impinging on the chip surface. Since the waveguide is not directly exposed to the gas jet, it produces only a gentle background velocity near the chip. The additional forced-convection heat loss is therefore negligible compared with conduction-dominated heat transfer.

### Numerical simulation

The photothermal transient time-domain profile and steady state frequency-domain simulation are investigated using the finite element method (FEM) with COMSOL Multiphysics. The gas flow in the evanescent region is considered as a continuum, following the physical laws of thermodynamics, diffusion, and thermal conductivity. Since the gas flow rate is relatively slow, thermal conduction is considered the main dissipation mechanism in this PT model, where convection and radiation are neglected. We use the thermal conductivity of air for the gas medium, the value of which is 0.026 W/(m ∙ K) at ambient temperature and is temperature-dependent. The limit that can optimize thermal conductivity through suspended design is that both the SiO_2_ and Si layers are completely removed (etched away, which is practically impossible), and the corresponding value of ${\kappa }_{{\rm{eq}}}$ is then 0.0344 W/(m ∙ K), only about 2.2 times larger than the actual suspended ChGW in this case. Another consideration is that the trace gas absorption is regarded as weak (*αΓCL*«1) so that the pump depletion along the waveguide can be safely ignored. For our case here, under conditions of *α* = 1.05 cm^-1^, *Γ* = 95%*, C* = 1% and *L* = 1.2 cm, the relative error is <1%, which satisfies the weak absorption condition.

## Supplementary information

Supporting Information

## Data Availability

The data that support the plots within this paper and other findings of this study are available from the corresponding authors upon reasonable request.
