# Supplementary material for: Suspended waveguide-enhanced near-infrared photothermal spectroscopy for ppb-level molecular gas sensing on a chalcogenide chip
Source: Light Sci Appl. 2026 Feb 17;15:116. doi: 10.1038/s41377-026-02196-7 (PMC12914002; doi:10.1038/s41377-026-02196-7)
Supplement: Supplementary file 1 — Supporting Information [file 41377_2026_2196_MOESM1_ESM.docx]

**Supplementary information for**

**Suspended waveguide-enhanced near-infrared photothermal spectroscopy for ppb-level molecular gas sensing on a chalcogenide chip**

Kaiyuan Zheng^1,2,†^, Hanyu Liao^1,2,†^, Fengbo Han^3,†^, Xueying Wang^4^, Yan Zhang^3^, Jiaxin Gu^3^, Pengcheng Zhao^1,2^, Haihong Bao^1,2^, Shaoliang Yu^3^, Qingyang Du^3*^, Lei Liang^5^, Chuantao Zheng^4*^, Wei Jin^1,2*^, and Lijun Wang^5^

^1^Department of Electrical and Electronic Engineering and Photonics Research Institute, The Hong Kong Polytechnic University, Hong Kong, 999077, China

^2^Photonics Research Center, The Hong Kong Polytechnic University Shenzhen Research Institute, Shenzhen, 518071, China

^3^Zhejiang Lab, Hangzhou, 311121, China

^4^State Key Laboratory of Integrated Optoelectronics, JLU Region, College of Electronic Science and Engineering, Jilin University, Changchun, 130012, China

^5^State Key Laboratory of Luminescence and Applications, Changchun Institute of Optics, Fine Mechanics and Physics, Chinese Academy of Sciences, Changchun 130033, China

^†^These authors contributed equally to this work

^*^Corresponding authors: qydu@zhejianglab.edu.cn, zhengchuantao@jlu.edu.cn, wei.jin@polyu.edu.hk

**Note 1. Theory of the suspended waveguide-enhanced PTS (SWE-PTS)**

The suspended waveguide enhances the performance of the photothermal spectroscopy (PTS) by improving the efficiency of two physical processes: the enhanced pump absorption that produces a more efficient heat source, and the enhanced heat accumulation near the waveguide region that results in higher PT phase modulation efficiency. These enhancements depend on the dimensions of the suspended waveguide as well as the pump and probe wavelengths. For simplicity, we ignore the surface roughness and assume waveguide is uniform along the *z*-axis.

(i) We first discuss the enhancement of pump absorbance that produces heat source. In general, for a monochromatic pump with its wavelength centered at the gas absorption line in free-space, the heat source is a time-invariance source that is equal to $\alpha CI_{p}$, $\alpha$ is the gas absorption coefficient, $C$ is the gas concentration, and $I_{p}(x,y)$ is the pump intensity. For the heat source in SWE-PTS, the pump is wavelength-modulated around the gas absorption line, and the light-gas interaction is actuated by the pump evanescent field in gas. Hence, the corresponding heat source is time-periodic and distributed outside the waveguide material. Similar to the wavelength modulation spectroscopy (WMS),^1^, the heat source can be decoupled as a sum of *n*^th^ order harmonic sources as:

$$\begin{aligned} Q\left( x,y,t \right)=\sum_{n} Q_{n}=\sum_{n} H_{n}\alpha CI_{p}^{\mathrm{gas}}(x,y)e^{jn\omega t}\#\left( S1 \right) \end{aligned}$$

here $H_{n}$ is the harmonic modulation coefficient, $\alpha$ is the peak absorption coefficient of the selected gas absorption line, $C$ the concentration of the gas, $\omega$ the modulation frequency of the pump beam. The $I_{p}^{\mathrm{gas}}$ used here is the intensity of the pump beam with the superscript denotes the gas region, which is defined through:

$$\begin{aligned} I_{p}^{\mathrm{gas}}\left( x,y \right)=\frac{1}{2}c\varepsilon_{0}n_{\mathrm{gas}}\left| E_{p}\left( x,y \right) \right|^{2}\#\left( S2 \right) \end{aligned}$$

here *c* is the speed of light, $\varepsilon_{0}$is the dielectric constant, $n_{\mathrm{gas}}$ is the refractive index of the gas material, $E_{p}\left( x,y \right)$ is the pump electric field. For gas absorption line in the profile of Lorentz function, the WMS technique picks the second order harmonic signal $n=2$ with $H_{2}=0.343$ at optimized modulation parameters. Hence, we focus on the second harmonic heat source $Q_{2}$. To compare the heat generation efficiency among different waveguide structures, we introduce the heat source power $P_{Q}$, which is defined as:

$$\begin{aligned} P_{Q}= \iint_{\mathrm{total}} Q_{2} dxdy\#\left( S3 \right) \end{aligned}$$

here $P_{Q}$ is in the unit of Watt that denotes the energy transferred from pump to thermal energy. Combine Eqs. (S1-S3), the heat source power is related to the intensity of the pump evanescent field as:

$$\begin{aligned} P_{Q}=H_{2}\alpha Ce^{j2\omega t}\iint_{\mathrm{gas}} I_{p}^{\mathrm{gas}} dxdy\#\left( S4 \right) \end{aligned}$$

Since $I_{p}^{\mathrm{gas}}$ is only a fraction of the total pump intensity, it is convenient to express the integration of $I_{p}^{\mathrm{gas}}$ by using the measurable pump power $P_{Q}$ through:

$$\begin{aligned} \iint_{\mathrm{gas}} I_{p}^{\mathrm{gas}} dxdy=P_{p}\Gamma\#\left( S5 \right) \end{aligned}$$

Here the variable $\Gamma$ is some fraction that scales the heat source power to the pump power, which is exactly the gas confinement factor (GCF)^2^. The definition of $\Gamma$ immediately follows Eq. (S5) as:

$$\begin{aligned} \Gamma:=\frac{\iint_{\mathrm{gas}} I_{p}^{\mathrm{gas}} dxdy}{P_{p}}=\frac{n_{\mathrm{gp}}\iint_{\mathrm{gas}} \varepsilon\left( x,y \right)\left| E_{p}\left( x,y \right) \right|^{2} dxdy}{n_{\mathrm{gas}}\iint_{\mathrm{total}} \varepsilon\left( x,y \right)\left| E_{p}\left( x,y \right) \right|^{2} dxdy}\#\left( S6 \right) \end{aligned}$$

here $n_{\mathrm{gas}}$ is refractive index (RI) of gas, and we have used the definition of $n_{\mathrm{gp}}=cU_{p}/P_{p}$ in deducting Eq. (S6), where $U_{p}$ is the pump energy stored in the fiber cross-section. Based on Eq. (S5) and Eq. (S6), one can define the normalized pump field distribution $\psi_{p}$ as:

$$\begin{aligned} \psi_{p}=\frac{\sqrt{\varepsilon_{0}}n(x,y)E_{p}\left( x,y \right)}{\iint_{\mathrm{total}} \varepsilon_{0}n\left( x,y \right)^{2}\left| E_{p}\left( x,y \right) \right|^{2} dxdy}\#\left( S7 \right) \end{aligned}$$

$$\begin{aligned} \Gamma=\frac{n_{\mathrm{gp}}}{n_{\mathrm{gas}}}\iint_{\mathrm{gas}} \psi_{p}^{*}\psi_{p} dxdy=\frac{n_{\mathrm{gp}}}{n_{\mathrm{gas}}}\gamma_{p}\#\left( S8 \right) \end{aligned}$$

here $\psi_{p}$ is simply the electric field $E_{p}$ normalized by its stored energy $U_{p}$, and we have also introduced the evanescent field ratio $\gamma_{p}$ representing the fraction of the pump intensity in the gas region:

$$\begin{aligned} \gamma_{p}:=\frac{\iint_{\mathrm{gas}} \psi_{p}^{*}\psi_{p} dxdy}{\iint_{\mathrm{total}} \psi_{p}^{*}\psi_{p} dxdy}\#\left( S9 \right) \end{aligned}$$

Combining Eq. (S4) with Eqs. (S4-S9), the power of the heat source can be expressed as:

$$\begin{aligned} P_{Q}=H_{2}\alpha CP_{p}\Gamma e^{j2\omega t}\propto\frac{n_{\mathrm{gp}}}{n_{\mathrm{gas}}}\alpha CP_{p}\gamma_{p}\#\left( S10 \right) \end{aligned}$$

hence the suspended waveguide enhances the power of the heat source in the PTS with a higher $\Gamma$. This enhancement attributes to a larger proportion of the evanescent field in the gas region. For the suspended waveguide used in this work, the GCF could be 3−4 times larger as compared to the non-suspended waveguide.

(ii) We discuss the enhancement of heat accumulation that improves photothermal phase modulation. The second-order harmonic heat sources $Q_{2}(x,y,t)$ results in a temperature change in the form of $\Delta T(x,y,t)=\tilde{T}(x,y)e^{-j2\omega t}$ through the heat conduction, which lead to subsequent probe phase modulation at the same frequency. Assuming that $\alpha CL\ll1$ for trace gas detection, $L$ is the length of the waveguide, the heat transfer equation can be solved in the form of linear perturbation. The resulting temperature change may be determined by solving the heat transfer equation for $\tilde{T}$ in the frequency domain:

$$\begin{aligned} Cj2\omega\tilde{T}-\nabla\cdot\left( \kappa\nabla\tilde{T} \right)+C\vec{v}\cdot\nabla\tilde{T}=\tilde{Q}_{2}\#\left( S11 \right) \end{aligned}$$

here $C=\rho c$ is the volumetric heat capacity with $\rho,c$ being the gas density and heat capacity,$\kappa$ is heat conductivity, $\vec{v}$ is the gas flow velocity which is set to be zero in solid region. The ~ denotes the amplitude of the variables in the frequency domain, *i.e.* $\tilde{Q}_{2}=H_{2}\alpha CI_{p}^{\mathrm{gas}}$. To analytically solve Eq. (S11), we need to make some assumptions first. Since the non-zero gas velocity $\vec{v}$ would improve the heat conduction and reduce the heat accumulation, in sensor fabrication the ChG waveguide is placed at the bottom of the gas chamber, approximately 3.5 mm below the jet axis of the gas inlet. By doing so, the local gas velocity near the waveguide is governed by the net through-flow rather than by the nozzle exit velocity, which is typically small and can be safely ignored. In our experiment, we employed 200 sccm inlet and outlet in a 1 cm×1 cm×0.5 cm gas cell. The cross-sectional area is $A_{cell}=5\times{10}^{-5}m^{2}$ and $F_{out}=200 sccm$. Away from the jet core, the background “sweep” velocity can be estimated by:

$$\begin{aligned} v\approx\frac{F_{out}}{A_{cell}}\approx6.7 cm/s\#\left( S12 \right) \end{aligned}$$

Whether convection significantly alters the heat dissipation is determined by the Péclet number:

$$\begin{aligned} Pe=\frac{vL_{e}\rho c}{\kappa}\#\left( S13 \right) \end{aligned}$$

with $L_{e}$ being the relevant thermal length scale. For heat transfer equation solved at frequency domain, the appropriate length scale is the thermal penetration depth as:

$$\begin{aligned} {L_{e}=\delta}_{i}=\sqrt{\frac{2\kappa_{i}}{\rho_{i}c_{i}\omega_{m}}}\#\left( S14 \right) \end{aligned}$$

At frequency $f=2\mathrm{kHz}$, the calculated Péclet number for air is $Pe \sim{10}^{-2}\ll1$, indicating that conduction dominates and convection has a negligible effect under our experimental conditions. Hence, the combination of off-axis geometry, small local velocities, and small thermal penetration depth at modulation frequencies of interest ensures that heat conduction is the governing mechanism. Besides, we neglected the interfacial thermal resistance (Kapitza resistance $R_{K}$) which is typically in the range of ${10}^{-5}-{10}^{-6} m^{2}K/W$, much smaller than the thermal resistance of ChG ($3.8\times{10}^{-6}m^{2}K/W$) and gas ($1.0\times{10}^{-3} m^{2}K/W$).

To get the analytic solution of $\tilde{T}$, we introduce an equivalent heat transfer model based on Eq. (S11). For a fixed modulation frequency and fixed pump wavelength (fixed $\psi_{p}$), increasing the power $P_{Q}$ does not affect the temperature field profile but linearly increases the temperature change amplitude at any arbitrary point. To show this, we decompose $\tilde{T}\left( x,y \right)$ into a product:

$$\begin{aligned} \tilde{T}\left( x,y \right)={T_{\mathrm{eq}}\psi}_{T}\left( x,y \right)\#\left( S15 \right) \end{aligned}$$

here $\psi_{T}(x,y)$ is spatial profile that captures the distribution’s geometric dependence, and $T_{\mathrm{eq}}$ is the scalable equivalent amplitude. The decomposition is valid since the fixed modulation frequency and pump wavelength define a specific eigen mode $\psi_{T}$ for the heat transfer system. The integration form of Eq. (S11) relates the temperature change $\tilde{T}$ to the heat source power defined by Eq. (S3) as:

$$\begin{aligned} -j2\omega_{m}T_{eq}\iint_{total} C\psi_{T}dxdy-T_{eq}\iint_{total} \nabla\cdot\left( \kappa\nabla\psi_{T} \right)dxdy=\iint_{\mathrm{total}} \tilde{Q}_{2} dxdy=P_{Q}\#\left( S16 \right) \end{aligned}$$

where we have substituted Eq. (S15) and neglected the heat convection term. In general, direct spatial integration of Eq. (S11) to derive Eq. (S16) imposes a fundamental constraint of global thermal energy balance. The operation is generally invalid without explicitly verifying boundary compatibility, since it requires the net heat flux through boundaries to exactly offset the integration source term. In SWE-PTS, however, such a balance is physically achievable as the boundaries permit heat exchange, e.g. under constant-temperature boundary conditions or in unbounded domains where the temperature field decays at infinity. In our model, we assume the boundary at constant temperature 298 K, hence Eq. (S16) is appliable. This assumption might be invalid under the case where the photonic chip could exchange heat with the surrounding gas via natural convection, or the inlet gas flow introduces forced advection. Whether the heat convection dominates the heat transfer process can be estimated by the Peclet number, which in our case (1 cm×1 cm×0.5 cm gas cell and 200 sccm gas inlet) is $P_{e}\sim{10}^{-2}\ll1$, hence the heat conduction dominates and convection is negligible.

The equivalent heat transfer model Eqs. (S15-S16) is obtained from spatial coordinate decomposition and direct integration. However, the $\psi_{T}$ here is a general temperature profile whose normalization condition has not been specified. For SWE-PTS, we are only interested in the overlap between the probe field $\psi_{b}$ and the temperature field. Thus, similar to the coupled-mode theory, we choose to normalize $\psi_{T}$ by projecting it onto the probe mode under a weighted inner product:

$$\begin{aligned} \iint_{\mathrm{total}} \psi_{b}^{*}\psi_{T}\psi_{b} dxdy:=1\#\left( S17 \right) \end{aligned}$$

This normalization ensures unit coupling strength between $\psi_{T}$ and the probe mode $\psi_{b}$ in SWE-PTS, i.e., the efficiency of temperature field $\psi_{T}$ coupled to phase modulation of $\psi_{b}$ is unit. It is now sufficient to deduce the mathematical form of $T_{\mathrm{eq}}$ by multiplying $T_{\mathrm{eq}}$ on both sides of Eq. (S17):

$$\begin{aligned} T_{\mathrm{eq}}=\iint_{\mathrm{total}} \psi_{b}^{*}\tilde{T}\psi_{b} dxdy\#\left( S18 \right) \end{aligned}$$

Eq. (S18) demonstrates the physical meaning of $T_{\mathrm{eq}}$ under our normalization condition Eq. (S17): $T_{\mathrm{eq}}$ is the equivalent temperature amplitude that is probed by the probe beam. From now on, $T_{\mathrm{eq}}$ is referred as probed temperature. Combining Eq. (S16) with Eq. (S18), it is also possible to define the equivalent volumetric heat capacity $C_{eq}$ and equivalent heat conductivity $\kappa_{\mathrm{eq}}$ that converts the heat source energy to the probed temperature:

$$\begin{aligned} C_{eq}:=\iint_{total} C\psi_{T}dxdy= \iint_{total} \rho c\psi_{T}dxdy\#\left( S19 \right) \end{aligned}$$

$$\begin{aligned} \kappa_{\mathrm{eq}}:=\iint_{\mathrm{total}} \nabla\cdot\left( \kappa\nabla\psi_{T} \right)dxdy\#\left( S20 \right) \end{aligned}$$

As shown in Eqs. (S19-S20), the defined $C_{eq}$ and t$\kappa_{eq}$ are exactly the volumetric heat capacity and heat conductivity experienced by the temperature field mode field $\psi_{T}$. As a result, the solution of the heat transfer equation is:

$$\begin{aligned} T_{\mathrm{eq}}=\frac{P_{Q}}{j2\omega_{m}C_{eq}-\kappa_{eq}}\#\left( S21 \right) \end{aligned}$$

Eq. (S19) is the general solution under demodulation frequency of $2\omega_{m}$. To achieve maximum photothermal phase modulation efficiency, we choose the demodulation frequency $\omega_{m}=1kHz$ which is much smaller than the 3 dB roll-off frequency. For this case, we have $j2\omega_{m}C_{eq}\ll\kappa_{eq}$. In this case, the thermal inertia term can be neglected and Eq. (S21) becomes a compact form:

$$\begin{aligned} T_{\mathrm{eq}}\approx-\frac{P_{Q}}{\kappa_{eq}}\#\left( S22 \right) \end{aligned}$$

Numerical calculation reveals that the error of using Eq. (S22) to replace Eq. (S21) at 1 kHz is only $0.5\%$. According to Eq. (S20), $\kappa_{\mathrm{eq}}$captures the whole thermal conduction characteristics on the waveguide cross-section plane $dxdy.$ For example, if the waveguide material is uniform and has a heat conductivity of $\kappa_{0}$, for a point source $Q_{2}$ with a low modulation frequency $\omega_{m}\ll\kappa_{eq}/2C_{eq}$ , Eqs. (S16, S20) yields:

$$\begin{aligned} \iint_{\mathrm{total}} \nabla^{2}\psi_{T} dxdy=1\#\left( S23 \right) \end{aligned}$$

$$\begin{aligned} \kappa_{\mathrm{eq}}= \iint_{\mathrm{total}} \nabla\cdot\left( \kappa_{0}\nabla\psi_{T} \right)dxdy=\kappa_{0}\#\left( S24 \right) \end{aligned}$$

where the equivalent heat conductivity $\kappa_{\mathrm{eq}}$ is exactly the material conductivity $\kappa_{0}$. Based on our equivalent heat transfer model, the probed temperature $T_{\mathrm{eq}}$ (equivalent temperature amplitude) can be expressed based on Eq. (S10) and (S22):

$$\begin{aligned} T_{\mathrm{eq}}=-\frac{H_{2}\alpha CP_{p}\Gamma}{\kappa_{\mathrm{eq}}} e^{j2\omega t}\#\left( S25 \right) \end{aligned}$$

Eq. (S25) demonstrates the enhancement provided by the suspended design. As compared to the non-suspended one, the suspension decreases the equivalent thermal conductivity $\kappa_{\mathrm{eq}}$, reducing heat dissipation and contributing to stronger heat accumulation, hence the temperature probed by the probe beam $T_{\mathrm{eq}}$ is larger.

(iii) The temperature change modulates the phase of the probe beam through thermo-optic effect and thermal expansion effect. The amplitude of the 2^nd^ harmonic phase modulation on the probe beam may be calculated based on first order approximation:

$$\begin{aligned} \Delta\varphi_{b}=\Delta\tilde{\varphi}_{b}e^{j2\omega t}=\frac{2\pi}{\lambda_{b}}\left( \bar{L}\Delta\tilde{n}_{b}+\bar{n}_{b}\Delta\tilde{L} \right)e^{j2\omega t}\#\left( S26 \right) \end{aligned}$$

here $\lambda_{b}$ is the wavelength of the probe beam, $\Delta\tilde{n}_{b}$ and $\Delta\tilde{L}$ are the probe mode RI change and the waveguide length change due to the temperature change. The “–” denotes the time averaged value of the variables. The temperature change modulates the probe mode index change $\Delta\tilde{n}_{b}$ through thermo-optic effect and thermo-photoelasticity effects by altering the permittivity of the material, while also modulates the waveguide length $\Delta\tilde{L}$ through thermal expansion effect by thermal strain. Probe mode index change $\Delta\tilde{n}_{b}$ is related to the permittivity change $\Delta\tilde{\varepsilon}_{r}$ through:

$$\begin{aligned} \Delta\tilde{n}_{b}=\sum_{i} \frac{n_{\mathrm{gb}}}{2n_{i}}\iint_{i} \psi_{b}^{*}\Delta\tilde{\varepsilon}_{ri}\psi_{b} dxdy\#\left( S27 \right) \end{aligned}$$

here $n_{\mathrm{gb}}$ is the group index of the probe mode, and the indices $i$ denote the target material (gas/ChG/SiO_2_/Si). The permittivity change due to the thermal-optic effect is expressed as:

$$\begin{aligned} \Delta\tilde{\varepsilon}_{ri}^{TOE}=2n_{i}\frac{dn_{i}}{dT}\tilde{T}\#\left( S28 \right) \end{aligned}$$

$$\begin{aligned} \Delta\tilde{\varepsilon}_{ri}^{PE}=-n^{4}\vec{p}_{i}:\vec{\tilde{S}}=-n^{4}\left[ \begin{matrix} \begin{matrix} p_{i11} & p_{i12} & p_{i12} \\ p_{i12} & p_{i11} & p_{i12} \\ p_{i12} & p_{i12} & p_{i11} \end{matrix} & \begin{matrix} 0 & 0 & 0 \\ 0 & 0 & 0 \\ 0 & 0 & 0 \end{matrix} \\ \begin{matrix} 0 & 0 & 0 \\ 0 & 0 & 0 \\ 0 & 0 & 0 \end{matrix} & \begin{matrix} p_{i44} & 0 & 0 \\ 0 & p_{i44} & 0 \\ 0 & 0 & p_{i44} \end{matrix} \end{matrix} \right]\left[ \begin{matrix} \begin{matrix} \tilde{S}_{xx} \\ \tilde{S}_{yy} \\ \tilde{S}_{zz} \end{matrix} \\ \begin{matrix} \tilde{S}_{zy} \\ \tilde{S}_{xz} \\ \tilde{S}_{yx} \end{matrix} \end{matrix} \right]\#\left( S29 \right) \end{aligned}$$

where we have employed $\Delta\tilde{\varepsilon}_{r}=2n\Delta\tilde{n}$ for deducting Eq. (S28), $dn_{i}/dT$ is the thermo-optic coefficient of the target material, $\vec{p}_{i}$ is the photoelasticity tensor of the target material, $\tilde{S}$ is the amplitude of the strain tensor oscillating at $2\omega_{m}$. As for the thermal expansion effect, the waveguide length change $\Delta\tilde{L}$ is related to the temperature change $\tilde{T}$ through:

$$\begin{aligned} \Delta\tilde{L}=\int_{L_{0}} \frac{\iint_{A} \mathcal{E}\frac{dL}{dT}\tilde{T} dxdy}{\iint_{A} \mathcal{E}dxdy}dz\#(S30) \end{aligned}$$

here $\mathcal{E}$ is the young’s modulus of the material, $L_{0}$ is the length of the waveguide under ambient temperature, and $dL/dT$ is the thermal expansion coefficient of the materials. The subscripts $A$ of the surface integrals denote the integration in the full waveguide cross-section region (gas/ChG/SiO_2_/Si).

The overall phase modulation is a summation of contributions from TOE, photoelasticity effect and thermal expansion effect as $\Delta\tilde{\varphi}_{b}=\Delta\tilde{\varphi}_{b}^{TOE}+\Delta\tilde{\varphi}_{b}^{PE}+ \Delta\tilde{\varphi}_{b}^{TEE}$, which are accordingly:

$$\begin{aligned} \Delta\tilde{\varphi}_{b}^{\mathrm{TOE}}=\frac{2\pi}{\lambda_{b}}L_{0}\Delta\tilde{n}_{b}^{TOE}\#\left( S31 \right) \end{aligned}$$

$$\begin{aligned} \Delta\tilde{\varphi}_{b}^{\mathrm{PE}}=\frac{2\pi}{\lambda_{b}}L_{0}\Delta\tilde{n}_{b}^{PE}\#\left( S32 \right) \end{aligned}$$

$$\begin{aligned} \Delta\tilde{\varphi}_{b}^{\mathrm{TEE}}=\frac{2\pi}{\lambda_{b}}n_{b}\Delta\tilde{L}\#\left( S33 \right) \end{aligned}$$

The value of these terms can be numerically solved by combing Eqs. (S31-33) with Eqs. (S27-30). Typically, photoelasticity is extremely weaker than the others since the photoelasticity coefficient is two or three orders of magnitude smaller than the thermo-optic and thermal expansion coefficient. Besides, since the suspended waveguide structure is a multilayer structure, the axial thermal expansion of the waveguide core (ChG) is constrained by mechanical compatibility across other bonded layers (SiO_2_/Si). Consequently, the thermal expansion efficiency is much lower than that of an isolated, freely expanding ChG waveguide and depends on layer stiffnesses and boundary conditions. Fig. S1 shows the numerically calculated $\Delta\tilde{\varphi}_{b}^{\mathrm{TOE}}, \Delta\tilde{\varphi}_{b}^{\mathrm{PE}}$ and $\Delta\tilde{\varphi}_{b}^{\mathrm{TEE}}$, where the relative effort of neglecting the photoelasticity and thermal expansion effect is <0.1%. Hence, the PT phase modulation can be approximated only considering the thermo-optic effect in Eq. (S27-28). Combining Eq. (S27-28) and Eq. (S31), we can solve the probe mode index change $\Delta\tilde{n}_{b}$ as:

$$\begin{aligned} \Delta\tilde{n}_{b}=\frac{n_{\mathrm{gb}}}{n_{\mathrm{ChG}}}\frac{dn_{\mathrm{gas}}}{dT}\iint_{\mathrm{ChG}} \psi_{b}^{*}\tilde{T}\psi_{b} dxdy+\frac{n_{\mathrm{gb}}}{n_{\mathrm{gas}}}\frac{dn_{\mathrm{gas}}}{dT}\iint_{\mathrm{gas}} \psi_{b}^{*}\tilde{T}\psi_{b} dxdy\#\left( S34 \right) \end{aligned}$$


**Fig. S1 Phase modulation efficiency** $\boldsymbol{k}^{\boldsymbol{*}}$ **due to thermo-optic effect (TOE), photoelasticity (PE) and thermal expansion effect (TEE).** The phase modulation efficiency is calculated under 100 mW pump power and 100 ppm acetylene for different buffer zone thickness $h_{2}$.

Following Eq. (S34), we can define an equivalent thermo-optic coefficient $e_{\mathrm{eq}}^{\mathrm{TO}}$ for the waveguide structure:

$$e_{\mathrm{eq}}^{\mathrm{TO}}=\frac{\left( \frac{1}{n_{\mathrm{ChG}}}\frac{dn_{\mathrm{ChG}}}{dT}\iint_{\mathrm{ChG}} \psi_{b}^{*}\tilde{T}\psi_{b}dxdy+\frac{1}{n_{\mathrm{gas}}}\frac{dn_{\mathrm{gas}}}{dT}\iint_{\mathrm{gas}} \psi_{b}^{*}\tilde{T}\psi_{b}dxdy \right)}{\iint_{\mathrm{total}} \psi_{b}^{*}\tilde{T}\psi_{b}dxdy}$$

$$\begin{aligned} =\frac{1}{T_{\mathrm{eq}}}\frac{e_{\mathrm{ChG}}^{\mathrm{TO}}}{n_{\mathrm{ChG}}}\iint_{\mathrm{ChG}} \psi_{b}^{*}\psi_{T}\psi_{b}dxdy-\frac{1}{T_{\mathrm{eq}}}\frac{e_{\mathrm{gas}}^{\mathrm{TO}}}{n_{\mathrm{gas}}}\iint_{\mathrm{gas}} \psi_{b}^{*}\psi_{T}\psi_{b}dxdy\#\left( S35 \right) \end{aligned}$$

here $e_{ChG,gas}^{\mathrm{TO}}=dn_{ChG,gas}/dT$ is the thermo-optic (TO) coefficient of waveguide material and gas material. Based on Eq. (S31), (S34) and (S35), we can express the probe phase modulation as a function of $T_{eq}$ as:

$$\begin{aligned} \Delta\varphi_{b}\approx\frac{2\pi n_{\mathrm{gb}}L}{\lambda_{b}}T_{\mathrm{eq}}e_{\mathrm{eq}}^{\mathrm{TO}}e^{j2\omega t}=-\frac{2\pi n_{\mathrm{gb}}L}{\lambda_{b}}\frac{H_{n}\alpha CP_{p}\Gamma}{\kappa_{\mathrm{eq}}}e_{\mathrm{eq}}^{\mathrm{TO}}e^{j2\omega t}\#\left( S36 \right) \end{aligned}$$

Our approximation leads to a linear dependence of the probed temperature $T_{\mathrm{eq}}$on the power of the heat source $P_{Q}$, which is consistent with the experimental result shown in Fig. 6(c) of the Main Manuscript.

As depicted by Eq. (S36), the probe phase modulation $\Delta\varphi_{b}$ and hence the PTS signal depends on the dimensions of the core-layer structure via heat conduction $\kappa_{\mathrm{eq}}$ and hence probed temperature $T_{\mathrm{eq}}$, as well as the core-layer material that decides the thermo-optic coefficient $e_{\mathrm{eq}}^{\mathrm{TO}}$. To compare the PTS performance among different suspended geometric parameters, we define a normalized PT phase modulation efficiency as:

$$\begin{aligned} k^{*}\left( w,h,k \right):=\frac{\Delta\varphi_{b}}{\alpha CP_{p}L}\propto\Gamma\frac{e_{\mathrm{eq}}^{\mathrm{TO}}}{\kappa_{\mathrm{eq}}}\#\left( S37 \right) \end{aligned}$$

We numerically calculated the $k^{*}$ for different parameters using finite element method, and corresponding results are drawn in Fig. 2 of the Main Manuscript.

Note 2. On-chip Fabry-Pérot (F-P) cavity interferometer

The design principle of the F-P cavity is to achieve high fringe contrast, thereby enabling high phase-to-intensity conversion efficiency. This requires that the first reflection at the input facet and the second reflection from the output facet returning to the input facet have comparable power. Relevant parameters include the end-facet coupling loss, transmission loss, reflections between the interface of air/waveguides, and the waveguide length. To achieve this, we employed end-face polishing to minimize scattering at the waveguide facets. Tapered fibers with small mode-field diameter (~500 nm) were carefully aligned to the waveguide facets to reduce the coupling loss. In addition, a two-step patterning process was applied to fabricate the low-loss suspended ChGW. Fig. S2 illustrates the schematic and configuration of an on-chip F-P cavity interferometer. The reflection of the probe beam at two interfaces are governed by the Fresnel reflection law, which are formed between the air (*n*=1) and ChGW (*n*=2.7) with *R*_1_ = *R*_2_ ≈ 21%. The optimization of the waveguide length is demonstrated in Supplementary Note 3.


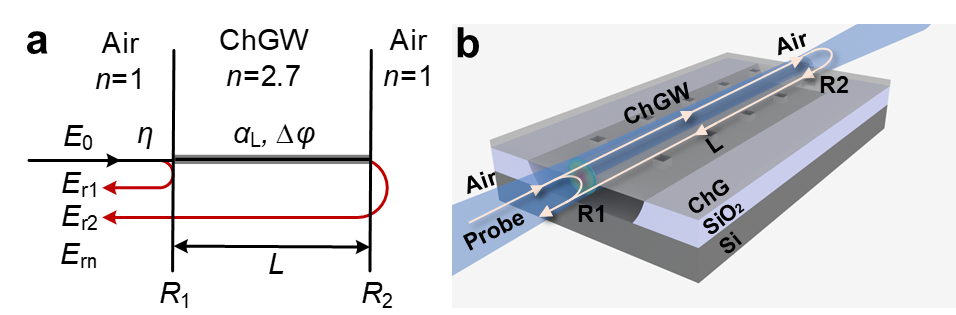


**Fig. S2 On-chip F-P cavity model.** **(a)** This model uses inherent Fresnel reflections between the interface of air/waveguides. $\eta$ is the end-facet coupling loss, $\alpha_{L}$ the transmission loss along the ChGW length $L$, $\Delta\varphi$ the probe phase modulation. **(b)** Schematic of the F-P cavity interferometer with suspended ChGW.

Assuming the input probe electric field is $E_{0}$, the reflected field at the first interface can be represented as$E_{r1}=E_{0}\sqrt{R_{1}\eta}$. The transmitted probe beam undergoes coupling loss ($\eta$), transmission loss ($\alpha$), and PT-induced phase modulation $\Delta\varphi$. After traversing the waveguide length $L_{\mathrm{WG}}$, the light reflects a second time as$E_{r2}=E_{0}(1-R_{1})\sqrt{R_{2}\eta}e^{2\alpha L}e^{j\varphi}$. While more reflections ($E_{\mathrm{rn}}$) could occur, losses beyond the third reflection become large enough, thus such higher-order reflections are generally negligible. Consequently, the reflected signal resulting from the F-P cavity can be described as

$$I=\left( E_{r1}+E_{r2} \right)\left( E_{r1}+E_{r2} \right)^{*}=E_{0}^{2}R_{1}\eta+E_{0}^{2}m^{2}+{2E}_{0}^{2}\sqrt{R_{1}\eta}me^{j\varphi} \left( S38 \right)$$

where *m* is defined as $m=(1-R_{1})\sqrt{R_{2}\eta}e^{2\alpha L}$. Here the phase difference $\varphi$ between the two reflected beams is expressed as

$$\varphi=\frac{4\pi n_{\mathrm{gb}}L_{\mathrm{WG}}}{\lambda_{b}}{+\varphi}_{0} (S39)$$

here $\varphi_{0}$ is the initial phase of the suspended ChGW. The fringe contrast $\upsilon$ can be expressed as

$$\upsilon=\frac{I_{\max}-I_{\min}}{I_{\max}+I_{\min}}=\frac{2m\sqrt{R_{1}\eta}}{R_{1}\eta+m^{2}} (S40)$$

Assuming a sinusoidal phase modulation $\varphi=\Delta\varphi sin(\omega t){+\varphi}_{0}$ with pump modulated angular frequency *ω*, the normalized interferometric signal can be expressed in the Bessel function as:

$$I_{\mathrm{norm}}=R_{1}\eta+m^{2}+2m\sqrt{R_{1}\eta} \{cos(\varphi_{0})[J_{0}\left( \Delta\varphi\right)+2\sum_{n=1}^{\infty} J_{2n}\left( \Delta\varphi\right)cos(2n\omega t)]$$

$$\begin{aligned} +sin(\varphi_{0})[2\sum_{n=0}^{\infty} J_{2n+1}\left( \Delta\varphi\right)\sin(\left( 2n+1 \right)\omega t)]\} \#\left( S41 \right) \end{aligned}$$

where $J_{n}\left( \Delta\varphi\right)$ is the Bessel function of order *n* with argument of $\Delta\varphi$. In this manner, the phase modulation could be converted into intensity output of the interferometer after the photodetector. The amplitude of $\Delta\varphi$ is always of main interest. If we consider Eq. (S41) with weak absorption approximation: $\Delta\varphi$*<<1, J*_0_ = 1*, J*_1_($\Delta\varphi$) = $\Delta\varphi$/2, the reflected intensity could be simplified as:

$$I_{\mathrm{norm}}=1+\frac{2m\sqrt{R_{1}\eta} sin(\varphi_{0})}{R_{1}\eta+m^{2}}\Delta\varphi\sin(\omega t) \left( S42 \right)$$

In this setup, the F-P cavity is working at a quadrature point, that is $\varphi_{0}$= *π*/2. The output intensity could be expressed as

$$I_{\mathrm{norm}}\left( t \right)=R_{1}\eta+m^{2}+2m\sqrt{R_{1}\eta} \Delta\varphi\sin(\omega t)=1+\upsilon\Delta\varphi\sin(\omega t) \left( S43 \right)$$

The PTS signal amplitude ($S_{\mathrm{PTS}}$) is defined as the 2*f*-demodulated voltage amplitude, which is proportional to the average probe power ($P_{\mathrm{avg}}$) at the PD, $\upsilon$, and $\Delta\varphi$, expressed as

$$S_{\mathrm{PTS}}\propto P_{\mathrm{avg}} \upsilon\Delta\varphi\left( S44 \right)$$

**Note 3. Optimization of the suspended waveguide length**

The accumulated PT-induced phase modulation $\Delta\varphi$ generally increases with the waveguide length *L*. However, with increasing *L*, the fringe contrast $\upsilon$ of such an F-P cavity also diminishes as the intensity of the light reflected from the second end of the ChGW decreases due to increasing transmission loss$\alpha_{L}$ along the waveguide, resulting in the degradation of *S*_PTS_. Therefore, there is a trade-off between $\Delta\varphi$ and $\upsilon$ with an optimal chip length *L*. As shown in Fig. S3(a), the transmission loss of the suspended ChGW is measured to be 2.6 dB/cm. The power *P* along the waveguide length *L* is expressed as

$$\log_{10}\frac{P(L)}{P_{0}}=\frac{{\alpha_{L}\cdot L|}_{\mathrm{dB}}}{10} \left( S45 \right)$$

here *P*_0_ is the initial power coupled into the suspended ChGW. The normalized fringe contrast $\nu_{\mathrm{norm}}$ is in the form of:

$$\nu_{\mathrm{norm}}=\frac{2m\sqrt{R_{1}\eta}}{R_{1}\eta+m^{2}}=\frac{2\sqrt{R_{1}\eta}\left( 1-R_{1} \right)\sqrt{R_{2}\eta}e^{2\alpha_{L}L}}{R_{1}\eta+{[\left( 1-R_{1} \right)\sqrt{R_{2}\eta}e^{2\alpha_{L}L}]}^{2}} (S46)$$

The PT phase modulation $\Delta\varphi$ could be calculated as a function of *l*:

$$\Delta\varphi=\frac{2\pi}{\lambda_{b}}\int_{0}^{L} k^{*}P\left( L \right)dl \left( S47 \right)$$

According to Eq. (S45), the $S_{\mathrm{PTS}}$ is proportional to the product of $\Delta\varphi$ and $\upsilon$. The results of $\Delta\varphi$, $\upsilon$ and their combined effect on $S_{\mathrm{PTS}}$ in terms of *L* is presented in Fig. S3(b). Calculations indicate that the optimal waveguide length for achieving the maximum PTS signal is approximately 0.8 cm, maintaining over 80% of the maximum value within the 0.5−1.4 cm range. Although the optimal waveguide length is 0.8 cm, we need to encapsulate it within a gas chamber and provide space for the gas inlet and outlet. As a result, we select a slightly longer length of 1.2 cm. At this length, the PTS performance still maintain about 90% of that at the optimal length. Additionally, a portion of the waveguide extends beyond the gas chamber to facilitate easier fiber coupling. The effective waveguide length interacting with gas inside the chamber is ~0.8−1 cm.


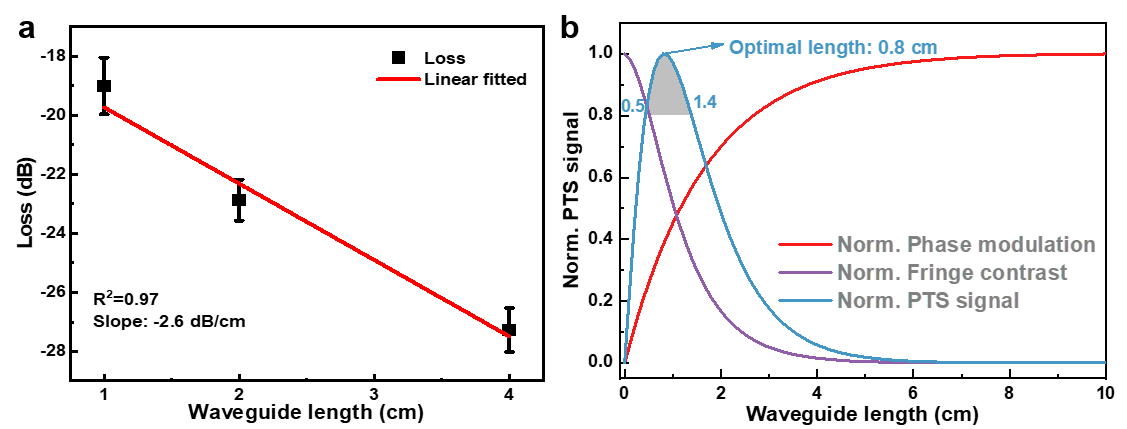


**Fig. S3 Suspended ChGW loss assessment and length optimization. (a)** Measured transmission loss of the suspended ChGW using different waveguide lengths. **(b)** Optimization of the suspended ChGW length to achieve the maximum PTS signal amplitude.

**Note 4. Comparison between suspended and non-suspended ChGW**

For the suspended ChGW, COMSOL Multiphysics is utilized for numerical simulation with parameters designed as *w*=1000 nm, *h*=300 nm, *k*=0.5, as shown in Fig. S4(a). Here suspended geometric parameters includes the rib width (*w*), height (*h*) and rib ratio (*k*). The heat source distributions are shown in Fig. S4(b) with different photothermal dynamics under varying demodulation frequencies. As demodulation frequency *f* increases, a concentration of heat towards the core layer with a corresponding decrease in the heat field is observed. This reduction is mainly attributed to the decreased heat generation within a modulation period. Fig. S4(c) depicts the probed temperature change in heat production in time domain, showing a decrease in steady-state temperature change from 13.7 to 1.26 mK as frequency increases from 2 to 200 kHz.


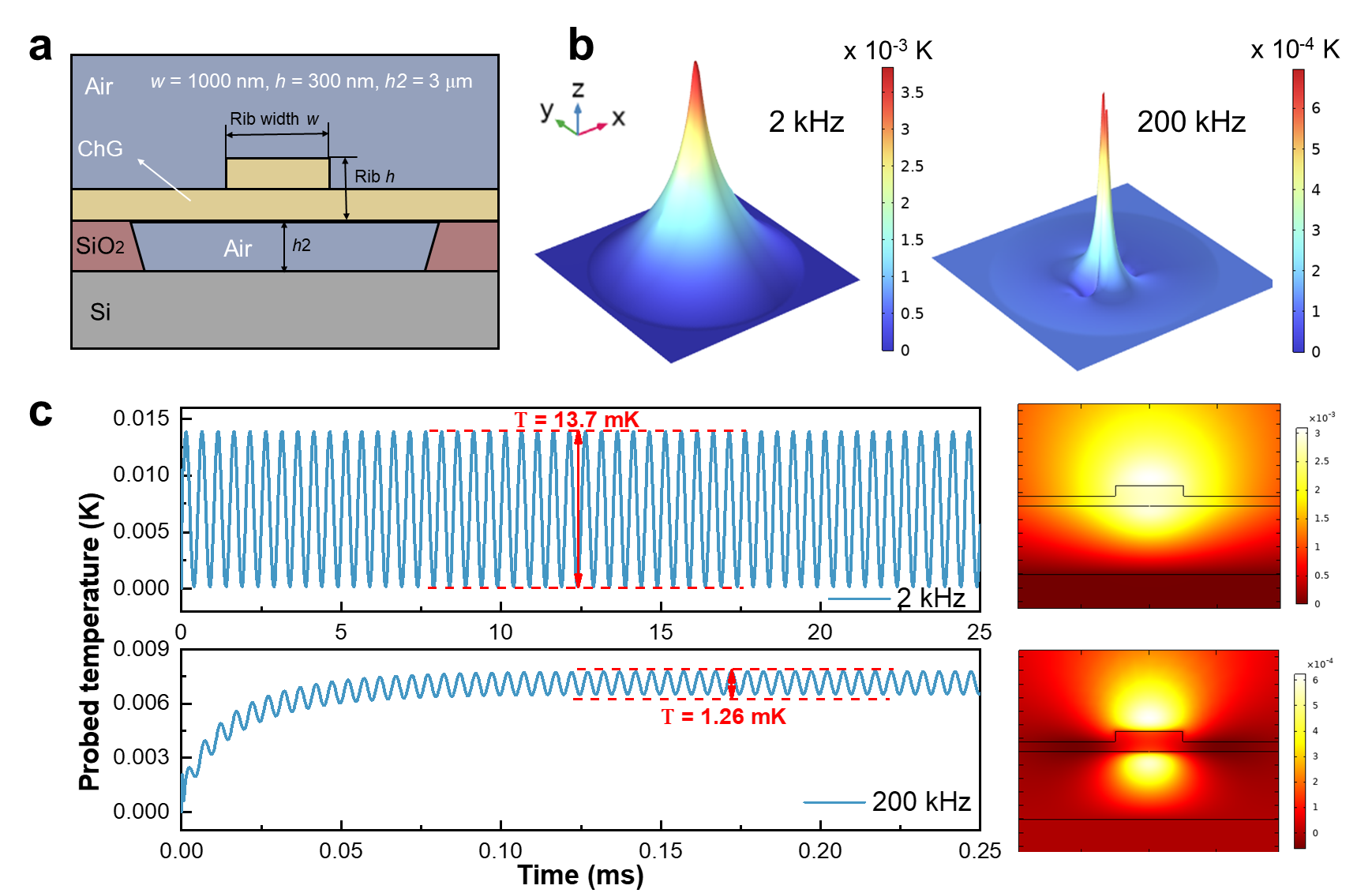


**Fig. S4 Suspended ChGW PT dynamics.** **(a)** Suspended ChGW model and **(b)** heat source profile with different demodulation frequency (2 and 200 kHz), respectively*.* **(c)** Dynamics of the heat conduction in time domain with corresponding steady-state thermal profiles.

Following a similar analytical approach in Fig. S4, we investigate the PT dynamics of non-suspended ChGW using parameters specified as *w*=1000 nm, *h*=300 nm, *k*=1 with SiO_2_ as the bottom cladding, not the air. As shown in Fig. S5(a). The heat source profiles with varying *f* are shown in Fig. S5(b), while Fig. S5(c) represent the time-domain dynamics, and the steady-state probed temperature decreases from 0.33 to 0.21 mK as *f* increases from 2 to 200 kHz.


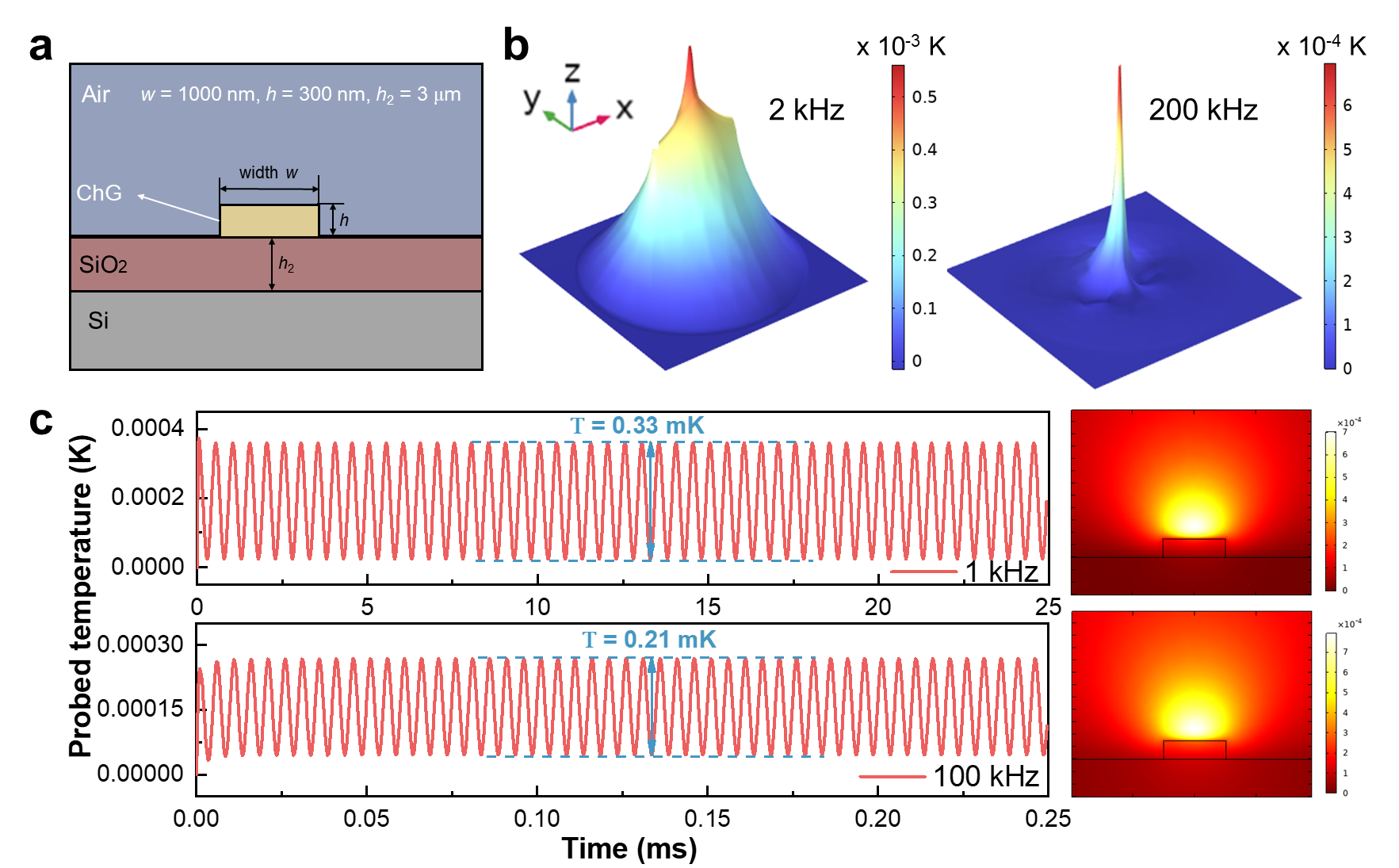


**Fig. S5 Non-suspended ChGW PT dynamics. (a)** Non-suspended ChGW model and **(b)** heat source profile with different demodulation frequency (2 and 200 kHz), respectively*.* **(c)** Dynamics of the heat conduction in time domain with corresponding steady-state profiles.

Detailed performance comparisons between these two waveguides are provided in Table S1. It is clearly seen that the GCF ($\Gamma$) of the suspended waveguide is nearly 4 times larger than that of the non-suspended one, yet it exhibits 10.6 times slower heat conduction and 1.08 times larger equivalent thermo-optic coefficient. Moreover, the heat source-induced probed temperature $T_{\mathrm{eq}}$ of the suspended waveguide is ~42 times larger than that of the non-suspended. The overall *k*^*^ enhances 45 times at 2 kHz, which further verifies the suspended ChGW a better option for on-chip PTS than traditional non-suspended strip one.

**Table S1** Detailed comparisons of the suspended- and non-suspended waveguide

| **Waveguide**  **structures** | $\boldsymbol{\Gamma}$  **(%)** | $\boldsymbol{\kappa}_{\mathbf{eq}}$  **(W/(m∙K))** | $\boldsymbol{e}_{\mathbf{eq}}^{\mathbf{TO}}$  **(1/K)** | $\boldsymbol{T}_{\mathbf{eq}}$  **(mK)** | ***k*^*^ (10^-6^ rad cm ppm^-1^ mW^-1^ m^-1^)**  **@2/20/200 kHz** | |
| --- | --- | --- | --- | --- | --- | --- |
| **Suspended** | 95 | 0.075 | 3.32$\times$10^-6^ | 4.71 | 19/9.3/1.7 |  |
| **Non-suspended** | 24 | 0.793 | 3.07$\times$10^-6^ | 0.112 | 0.42/0.4/0.24 |  |
| **Enhancement** | **4** | **10.6** | **1.08** | **42** | **45/23/7** |  |

**Note 5. Suspended ChGW fabrication**

The suspended ChGW core layer comprises a bottom slab and a top rib waveguide. The ratio of the thickness of the bottom slab to the rib waveguide should be more than 1:4 to ensure its robustness and stability. The fabrication of suspended waveguide is more complex than traditional channel waveguide, which requires two DUV patterning. Introducing a silicon oxide intermediate layer is more advantageous for removing photoresist from ChGW. In addition, the process involves the use of HF wet etching techniques to remove the underlying silicon oxide layer, demanding high precision to prevent ChGW from crack.

We utilize a 3-μm-thick thermally oxidized silicon wafer, a material that is harder and reacts more slowly with HF than other CVD-grown silicon oxides, aiding in the precise control of the etching rate. As shown in Fig. S6, the fabrication process is divided into two stages: initially, after depositing the required ChG and SiO_2_ films on the wafer, the first pattern is performed to create micro-holes for HF wet etching. The square holes, measuring 3×3 μm^2^ and spacing 7 μm apart, are patterned by using a Canon DUV stepper lithography system, followed by ICP etching. The structure is cleaned using NMP (1-methyl-pyrodine) solvent and IPA, then remove the silica intermediate layer with HF, and finally ultrasonically clean for 30 s to remove the residue completely. The second stage involves the precise alignment and patterning of the ChG rib waveguide over the first layer. DUV alignment error <40 nm ensures high precision of secondary lithography, the rib waveguides are etched to the designed thickness using ICP and then the photoresist is stripped, exposing the clean ChG rib waveguide. The final step involves immersing the sample in 5% HF, initiating wet etching of the thermally oxidized silicon through the micro-holes fabricated in the first stage. This etching process is meticulously timed and controlled to prevent over-etching and structural damage. After processing, the samples are soaked and rinsed in DI water to completely remove any residual HF.

**
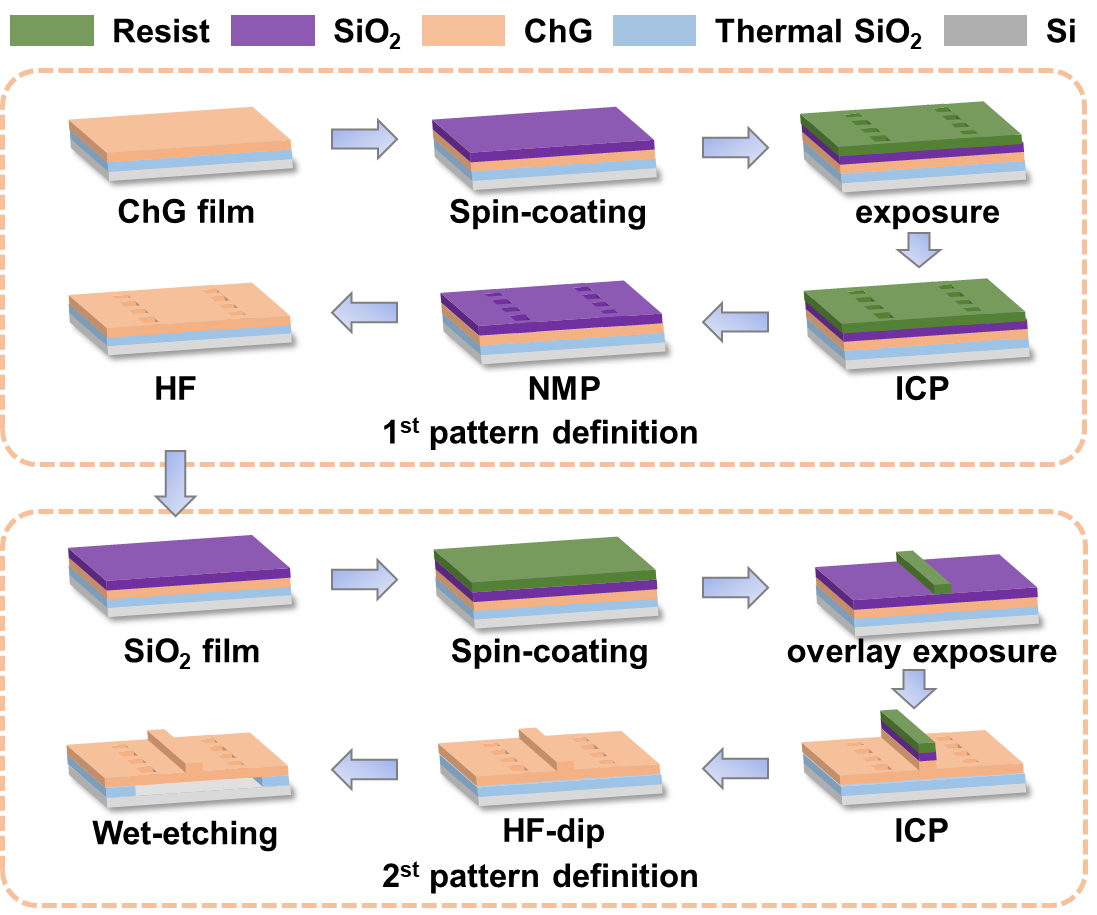
**

**Fig. S6 Fabrication process of suspended ChGW with two-step patterning process.** ICP: inductive-coupled plasma, NMP: 1-methyl-pyrodine.

**Note 6. Suspended ChGW characterization**

As shown in Figs. S7(a)-(c), controlling the wet-etching time allows for precise management of the lateral and vertical dimensions of the etched silicon oxide. As the HF etching time increases, the 3-μm-thick silicon layer beneath the ChG waveguide is more thoroughly removed, eliminating the silicon in the vertical direction while gradually increasing the lateral etching depth. Particularly, at an etching time of 50 min, the silicon oxide under the suspended waveguide is entirely removed. The integrity of the structure can be confirmed by scanning electron microscope (SEM) images. Inset of Fig. S7(c) includes a microscopic image of the suspended structure, visually illustrating the etching effects. Due to differences in transparency between the etched and unetched areas, the sides of the waveguide appear lighter, making the regions near the membrane and micro-holes visibly brighter than other areas, which is consistent with SEM observations. The CMOS-compatible process using a two-step stacked DUV lithography method ensures the accuracy of the pattern, and the design of the rib waveguide structure size endows the suspended waveguide with excellent structural stability.


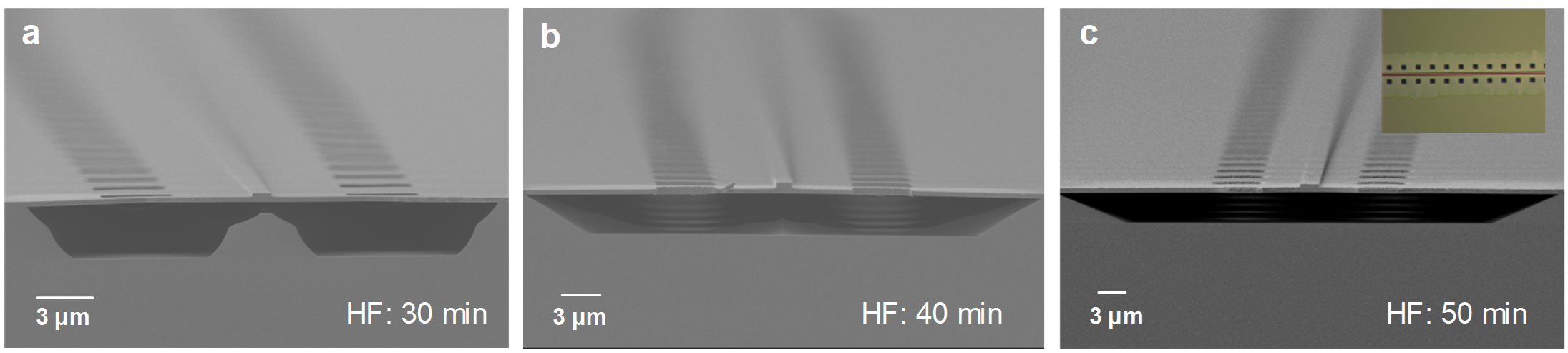


**Fig. S7 Suspended ChGW characterization.** SEM image under different etching time **(a)** 30 min **(b)** 40 min **(c)** 50 min. Inset is a microscopic top view of the suspended ChGW.

Since the quality of the film directly impacts the waveguide loss, it is hence crucial to monitor and characterize the film performance. A Zeiss SEM is used to capture surface and thickness images, while an energy-dispersive spectrometer (EDS) analyzes the film’s chemical composition. An atomic force microscope (AFM) characterizes the film's roughness. The deposition rate significantly affects the film's quality. As shown in Fig. S8, under clean chamber conditions, the AFM and SEM images of the ChG films deposited at 25 Å/s are displayed in Fig. S8(a). At this rate, the films exhibit considerable roughness with an average roughness (*R*_q_) of 0.9 nm due to the rapid aggregation of gaseous molecules during the growth process, which leads to uneven surfaces. Reducing the current to decrease the deposition rate to 16 Å/s and 13 Å/s results in films with smoother surfaces, as shown in Figs. S8(b) and (c), with *R*_q_ values of 0.529 nm and 0.476 nm are obtained, respectively.

**Fig. S8** **ChG film characterization.** AFM and SEM images of films deposited at different rates of **(a)** 25 Å/s, **(b)** 16 Å/s, and **(c)** 13 Å/s.

We then conduct EDS analyses on wafer with deposition rates of 13 Å/s. Fig. S9 shows the EDS mapping for a Ge_28_Sb_12_Se_60_ film, revealing a uniform distribution of Ge, Sb, and Se elements. It also presents the energy spectrum analysis of the ChG film grown at 13 Å/s, where peaks corresponding to C, O, and Si are attributed to the substrate elements, and the most prominent peaks are due to Se, indicating its higher proportion in this film. Through composition analysis, the measured element ratios of Ge, Sb, and Se are almost consistent with theoretical values of 28:12:60.

**Fig. S9** **Ge_28_Sb_12_Se_60_ film characterization.** EDS image of the films deposited at rate of 13 Å/s.

RI is one of the crucial optical parameters of materials. In this work, an ellipsometer, which measures film thickness and optical constants of material films, is used for RI measurement. We employ a German Sentech ellipsometer with a testing wavelength range of 780 nm to 2500 nm. The Sellmeier dispersion model is used for fitting to ensure a fit accuracy better than 99.99%. The resulting RI curve is shown in Fig. S10, where a RI value of ~2.75 is obtained around 1.5 μm.


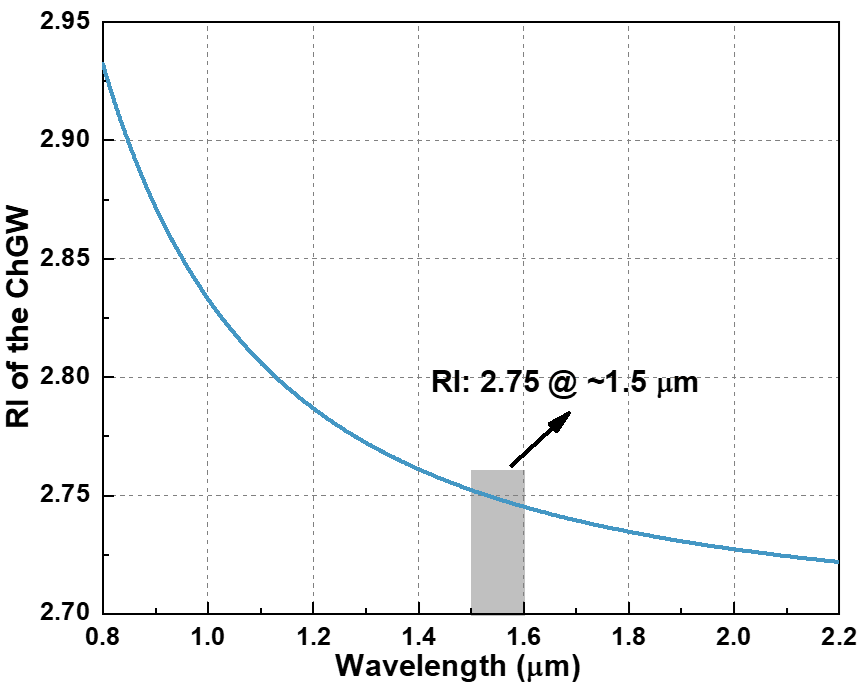


**Fig. S10** **Measured RI curve of the ChG film.** The wavelength range is from 0.8 to 2.2 μm.

**Note 7. Experimental investigation of the suspended ChGW**

The demonstrated ChGW could measure C_2_H_2_ gas at different absorption lines, as shown in Fig. S11, we coupled a broadband amplified spontaneous emission (ASE) source into the suspended waveguide and collected the transmitted beam using an optical spectrum analyzer (OSA). The C_2_H_2_ absorption spectrum was achieved after filling the pure C_2_H_2_ gas into the waveguide-integrated gas chamber. Each valley corresponded to different C_2_H_2_ absorption lines. Among them, the P(11) absorption line at 1531.58 nm was selected as the pump wavelength for the on-chip PTS, as it exhibited the strongest absorption coefficient in the P-branch of the near-infrared region. In addition, using pump lasers of different wavelengths, we could measure other absorption lines and gas species, as long as the pump wavelength falls within the waveguide's transmission range.

**Fig. S11** The transmission spectrum of ChGW after acetylene absorption.

Fig. S12 shows the reflection spectrum of the F-P interferometer around the pump and probe wavelengths. The excitation of the TM mode primarily relies on the offset alignment between the input fiber and the waveguide. By observing the interference fringes during the alignment process, we could determine whether the excitation is primarily TE or TM. After alignment, as the mode fields of TE and TM differ significantly, adjusting the polarization controllers (PCs) could not affect the pump/probe mode field and fringe spacing, but affect the fringe contrast.

The transmission loss of fundamental mode is small, resulting in larger fringe contrast, while higher-order modes have larger losses, leading to smaller contrast. As seen in Fig. 5(b), the excitation primarily involves the fundamental TM mode, with a very small contribution from higher-order modes. Although ChGW may support higher-order modes, they were not significantly excited in this case. Regarding the effects of higher-order modes on PT efficiency, higher-order pump modes are beneficial for PTS as they generate a larger evanescent field and higher heat source power, but may suffer from larger transmission losses. In contrast, higher-order probe modes should be avoided, as they experience weaker PT effects due to the thermo-optic effect being dominated by ChG rather than air. Higher-order modes have more optical power distributed in air, which results in lower PT efficiency. Additionally, these modes incur higher losses, leading to reduced fringe contrast.


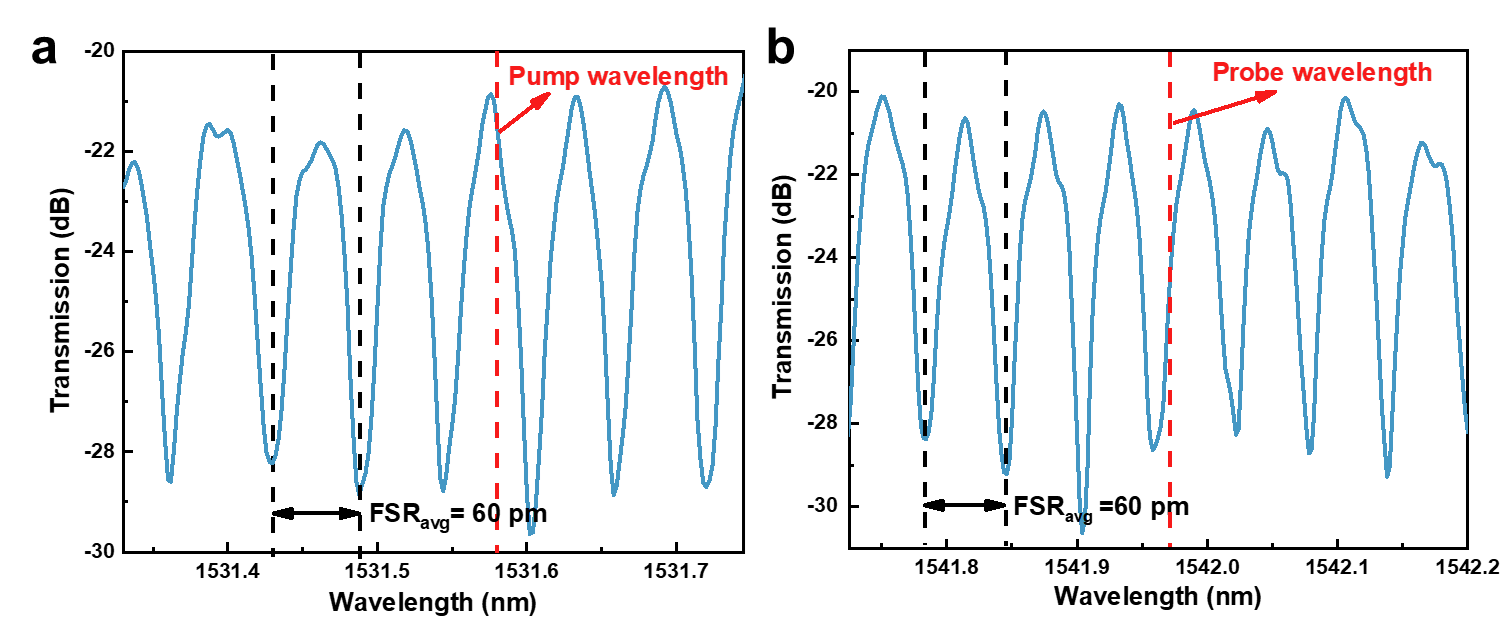


**Fig. S12** Reflection spectrum of the F-P cavity around the **(a)** pump and **(b)** probe wavelengths.

**Note 8. Numerical investigation of tracing different gases**

The principle of the SWE-PTS is to measure the heat generated by molecular absorption and the resulting phase modulation, which can be employed for tracing multiple gas species as long as the sensing platform supports multiple absorption wavelengths. We first calculated the leakage loss of the TM mode in the suspended ChGW over the wavelength range from ~1000 to ~3000 nm using the finite element method in COMSOL. We found in Fig. S13 that the fabricated ChGW supports low-loss transmission over the wavelength range from 1000 to 2500 nm. Therefore, we carried out photothermal simulations for some representative gas species with absorption in this wavelength range. We have simulated the photothermal phase modulation for four gas species, i.e., C_2_H_2_, CH_4_, CO and CO_2_ using pump wavelengths corresponding to their relatively strong absorption lines at 1.531 μm, 1.651 μm, 1.568 μm, and 2.004 μm, respectively. The thermo-optic and thermodynamic parameters used in the simulation are summarized in Table S2.

**Fig. S13** Simulated leakage loss of the suspended ChGW and the absorption cross-sections of the four gas species (C_2_H_2_, CH_4_, CO and CO_2_).

**Table S2** Parameters used in this simulation

| **Gas type** | **Wavelength (nm)** | **Rovib**  **mode** | $\boldsymbol{\alpha}$  **(cm^-1^)** | $\boldsymbol{\tau}_{\mathbf{re}}$  **(μs⋅atm)** | **Evanescent proportion**  **(**$\boldsymbol{\gamma}_{\mathbf{p}}$**)** | **Pump group index**  **(**$\boldsymbol{n}_{\mathbf{gp}}$**)** | **Gas confinement factor**  **(**$\boldsymbol{\Gamma}$**)** |
| --- | --- | --- | --- | --- | --- | --- | --- |
| C_2_H_2_ | 1531.58 | *v*_1_+*v*_3_ | 1.050 | 0.074 | 30.4% | 3.15 | 95% |
| CH_4_ | 1650.96 | 2*v*_3_ | 0.453 | 3.8 | 40.7% | 2.76 | 112% |
| CO | 1568.04 | *v*_3_ | 0.003 | 25 | 33.8% | 3.04 | 102% |
| CO_2_ | 2004.02 | 2*v*_1_+*v*_3_ | 0.142 | 11 | 45.6% | 2.19 | 99% |

Rovib mode: rovibrational mode of the molecule, $\alpha$: absorption coefficient, $\tau_{\mathrm{re}}$: relaxation time constant of the target rovibrational mode, $\Gamma$ is the gas confinement factor that scales the heat source power due to pump absorption, which is equal to ${n_{\mathrm{gp}}\gamma}_{p}$.

For trace gas detection, nitrogen is the dominant buffer gas. Therefore, the overall heat-transfer characteristics of the gas mixture do not change significantly. The main variations arise from the absorption-induced heat-source power $P_{Q}$ and its spatial distribution $\psi_{p}$, which is determined by the absorption coefficient, relaxation time, and pump wavelength. Using the parameters listed in Table S2, we performed numerical simulations of the photothermal response for each of the gas species at the specified absorption line. The calculated phase modulation amplitude at different modulation frequency is presented in Fig. S14. For different gas species, the relaxation time affects the frequency response at high frequencies, while the absorption coefficient and pump wavelength determines the maximum photothermal phase modulation that is approximately flat for frequency below 10 kHz. This demonstrates that the suspended waveguide can act as an efficient platform to generate photothermal phase modulation for all the four gas species, although the magnitude of phase modulation varies due to different absorption of the gas species. These results show that the same sensing platform can be used to detect different gas species by using a pump laser tuned to the absorption line of a specific gas molecule.

**Fig. S14** Calculated photothermal response for different gases. The gas concentrations are set to be 1% under 8 mW pump power and 1.2 cm ChGW length.

**Note 9. Performance comparison with other waveguide-based gas sensors**

Detailed sensing performance between the reported state-of-the-art waveguide gas sensors are shown in Table S3. Here we use the minimum detection limit (MDL) and noise equivalent absorption (NEA) to compare the detection sensitivities. Other parameters, including waveguide types with/without suspended structure, gas confinement factor (GCF), transmission loss, waveguide length, dynamic range and response time are also listed for comparison. The demonstrated SWE-PTS sensor yields an NEA of 3.8×10^-7^ cm^-1^, more than 1−4 orders of magnitude higher than that of the reported waveguide sensors. The dynamic range reaches nearly 6 orders of magnitude, more than 2 orders of magnitude larger than any of the previous on-chip gas sensing techniques. These comprehensive performances pave the way for high-sensitivity, high-selectivity, large dynamic range, fast-response, and ultracompact on-chip gas sensors.

**Table S3** Performance comparisons of the reported waveguide-based gas sensors

| **Method**  **& Ref.** | **Gas**  **&λ** | **Waveguide**  **types** | $\boldsymbol{\Gamma}$  **(%)** | **Loss**  **(dB/cm)** | **L**  **(cm)** | **MDL**  **@ time** | **NEA**  **(cm^-1^)** | **Dynamic range** | **Response time** |
| --- | --- | --- | --- | --- | --- | --- | --- | --- | --- |
| DAS^3^ | CH_4_  1650 nm | Si  Rectangular | 28.3 | 2 | 10 | 100 ppm  @60 s | 4.4×10^-5^ | >1.5×10^2^ | >400 s |
| DAS^4^ | CH_4_  3310 nm | ChG  Rectangular | 12.5 | 8 | 0.5 | 330 ppm | 1.3×10^-2^ | >1.5×10^2^ | NA |
| DAS^5^ | CH_4_  3310 nm | ChG  Rectangular | 10 | 7 | 2 | 4000 ppm | 1.6×10^-1^ | >2.5×10^2^ | NA |
| DAS^6^ | CH_4_  3270 nm | Si  Slot | 69 | 8.3 | 1.15 | 0.3 ppm @50 s | 1.5×10^-5^ | >3.3×10^3^ | >100 s |
| DAS^7^ | C_2_H_2_  2566 nm | Ta_2_O_5_  Suspended | 107 | 6.8 | 2 | 7 ppm  @25 s | 1.8×10^-4^ | >1.4×10^4^ | NA |
| DAS^8^ | CO_2_  4240 nm | Si  Suspended | 44 | 3 | 0.32 | 1000 ppm | 3.1×10^-1^ | >2.5×10^2^ | 2 s |
| DAS^9^ | CH_4_  3291 nm | ChG  Suspended | 112 | 4.5 | 1 | 30.3ppm  @43.4 s | 3.2×10^-4^ | >2.6×10^3^ | NA |
| WMS^10^ | C_2_H_2_  1532 nm | SU8  Rectangular | 1.7 | 3 | 13 | 28.7ppm  @48.6 s | 3×10^-5^ | >1.3×10^4^ | 3 s |
| WMS^9^ | CH_4_  3291 nm | ChG  Suspended | 112 | 4.5 | 1 | 5.9 ppm  @64.2 s | 6.2×10^-5^ | >1.3×10^4^ | NA |
| WMS^11^ | CH_4_  3291 nm | Si  Rectangular | 23 | 0.71 | 2 | 78 ppm  @0.2 s | 8.2×10^-4^ | >5×10^3^ | 3 s |
| WMS^12^ | CH_4_  3291 nm | ChG  Rectangular | 7.8 | 1.52 | 2 | 140 ppm  @32.4 s | 1.5×10^-3^ | >2.1×10^3^ | NA |
| RIS^13^ | NH_3_  1550 nm | Si  MRR | NA | NA | 5 μm (radii) | 5 ppm | NA | >1.6×10^2^ | >30 s |
| PTS^14^ | Resist  3419 nm | Si-MRR Suspended | NA | NA | 25μm (radii) | 5 ppm | 1.5×10^-4^ | >10^4^ | NA |
| PTS^15^ | C_2_H_2_  1531 nm | ChG  Rectangular | 10.5 | 2.5 | 2 | 4 ppm  @170 s | 4.5×10^-6^ | 1.4×10^5^ | NA |
| PTS^16^ | CO_2_  2004 nm | LN  Rectangular | 7.4 | 1.2 | 9.12 | 870 ppm  @190 s | 1.2×10^-4^ | >6.3×10^2^ | NA |
| **PTS**  **[This]** | **C_2_H_2_**  **1531 nm** | **ChG**  **Suspended** | **95** | **2.6** | **1.2** | **330 ppb**  **@65 s** | **3.8×10^-7^** | **9.1×10^5^** | **<1 s** |

DAS: direct absorption spectroscopy, WMS: wavelength modulation spectroscopy, a variation of DAS, RIS, reflective index spectroscopy, PTS: photothermal spectroscopy, Si: silicon, SU8: photoresist based on EPONSU-8 epoxy resin, ChG: chalcogenide glass, LN: lithium niobate, Resist: polymer photoresist AZ5214, MRR: micro-ring resonator, $\Gamma$: gas confinement factor, MDL: minimum detection limit with different averaging time, NEA: noise-equivalent absorption, NA: Not available.

**References**

1 Jin, W., Cao, Y., Yang, F. & Ho, H. L. Ultra-sensitive all-fibre photothermal spectroscopy with large dynamic range. *Nature Communications* **6**, 6767 (2015).

2 Robinson, J. T., Preston, K., Painter, O. & Lipson, M. First-principle derivation of gain in high-index-contrast waveguides. *Optics Express* **16**, 16659-16669 (2008).

3 Tombez, L., Zhang, E. J., Orcutt, J. S., Kamlapurkar, S. & Green, W. M. Methane absorption spectroscopy on a silicon photonic chip. *Optica* **4**, 1322-1325 (2017).

4 Su, P. *et al.* Monolithic on-chip mid-IR methane gas sensor with waveguide-integrated detector. *Applied Physics Letters* **114** (2019).

5 Han, Z. *et al.* On-chip mid-infrared gas detection using chalcogenide glass waveguide. *Applied Physics Letters* **108** (2016).

6 Yallew, H. D. *et al.* Sub-ppm methane detection with mid-Infrared slot waveguides. *ACS Photonics* **10**, 4282-4289 (2023).

7 Vlk, M. *et al.* Extraordinary evanescent field confinement waveguide sensor for mid-infrared trace gas spectroscopy. *Light: Science & Applications* **10**, 26 (2021).

8 Ottonello-Briano, F. *et al.* Carbon dioxide absorption spectroscopy with a mid-infrared silicon photonic waveguide. *Optics Letters* **45**, 109-112 (2019).

9 Pi, M. *et al.* Ultra-wideband mid-infrared chalcogenide suspended nanorib waveguide gas sensors with exceptionally high external confinement factor beyond free-space. *ACS Nano* **17**, 17761-17770 (2023).

10 Zhao, H. *et al.* WMS-based near-infrared on-chip acetylene sensor using polymeric SU8 Archimedean spiral waveguide with Euler S-bend. *Spectrochimica Acta Part A: Molecular Biomolecular Spectroscopy* **302**, 123020 (2023).

11 Zhao, H. *et al.* On-chip mid-infrared silicon-on-insulator waveguide methane sensor using two measurement schemes at 3.291 μm. *Frontiers in Chemistry* **10**, 953684 (2022).

12 Pi, M. *et al.* Theoretical and experimental investigation of on-chip mid-infrared chalcogenide waveguide CH4 sensor based on wavelength modulation spectroscopy. *Sensors Actuators B: Chemical* **362**, 131782 (2022).

13 Yebo, N. A. *et al.* Selective and reversible ammonia gas detection with nanoporous film functionalized silicon photonic micro-ring resonator. *Optics Express* **20**, 11855-11862 (2012).

14 Vasiliev, A. *et al.* On-chip mid-infrared photothermal spectroscopy using suspended silicon-on-insulator microring resonators. *ACS Sensors* **1**, 1301-1307 (2016).

15 Zheng, K. *et al.* Waveguide‐based on‐chip photothermal spectroscopy for gas sensing. *Laser & Photonics Reviews* **18**, 2301071 (2024).

16 Yan, Y., Feng, H., Wang, C. & Ren, W. On-chip photothermal gas sensor based on a lithium niobate rib waveguide. *Sensors Actuators B: Chemical* **405**, 135392 (2024).
